# A Review on Metal Ion Sensors Derived from Chalcone Precursor

**DOI:** 10.1007/s10895-022-02900-x

**Published:** 2022-02-24

**Authors:** Priyanka Mahesha, Nitinkumar S. Shetty, Suresh D. Kulkarni

**Affiliations:** 1 Department of Chemistry, Manipal Institute of Technology, Manipal Academy of Higher Education, Manipal, 576104 India; 2 Department of Atomic and Molecular Physics, Manipal Academy of Higher Education, Manipal, Karnataka 576104 India

**Keywords:** Chemosensor, Chalcone, Fluorescence, Pyrazoline, Metal ions

## Abstract

Disclosure of new molecular probes as chromogenic and fluorogenic cation sensors is scientifically exigent work. Recently chalcone derivatives gained more attention because of their structural variability. A suitable donor and acceptor groups separated by delocalized π-orbitals display excellent chromogenic and fluorogenic properties because of intramolecular charge transfer (ICT). These designed molecular frameworks provide the coordination sites to the incoming metal ions results in small changes in the optical properties. In a typical sensing behavior, coordination leads to a large conjugation plane with the probe resulted in hypo/hyperchromic shifts or red/blue shifts. In this review, we tried to converge the reported chalcone-derived sensors and explored the design, synthesis, metal ion sensing mechanism, and practical application of the probes. We expect that this review gives a basic outline for researchers to explore the field of chalcone-based sensors further.

## Introduction

Chalcones are a fundamental unit in various biologically potent compounds and have numerous applications such as anticancer [1, 2], anti-inflammatory [3], antioxidants [4], antimalarial [5], antiviral [6], anti-HIV [7], antiprotozoal [8], antimicrobial [9], antihypertensive [10], antifungal [11], antituberculosis [12], antidiabetic [13], antiulcer [14], antileishmanial [15], etc. In addition to their significance as potential pharmaceutical agents, chalcones are well known for their photochemical [16], optical [17], and Non-Linear Optical properties (NLO) [18] properties. Chalcones have been used as fluorophores in Organic Light-Emitting Diodes(OLED), chemosensors, and fluorescent probes [19, 20]. In addition to this, chalcones were also building blocks for the synthesis of the many heterocyclic compounds. Among the derivatives of chalcones, pyrazolines play a paramount role in the sensing field because of their distinctive structure. Pyrazolines were well known for their pharmacological properties, and also, the presence of many hetero atoms in the molecule induces excellent blue fluorescence. These emission properties were used in dye-sensitized based solar cells, optical emitting, hole transfer materials [21–24], etc.

Metal ions are present everywhere in living and non-living systems as beneficial metals and non-essential hazardous metals. E.g., essential metal ions such as iron, copper, zinc, and manganese are crucial for biological processes and enzymatic reactions [25], and other metal ions, such as gold, platinum, copper, etc., have anticancer activity [26]. But metal ions such as mercury, zinc, and cadmium hamper the living system and environment [27, 28]. In small amounts, metal ions such as zinc, iron, nickel, manganese, copper, cobalt, and gold are required for biological activity [29, 30]. Still, these are toxic to the body in excess amounts. E.g., the accumulation of lead causes arthritis, kidney and brain damage, etc., and mercury causes lung damage, memory problems, cancer, etc. The modern lifestyle increases all types of pollution in which water pollution is the primary one. This polluted water is threatening to all life in the environment. Most of the industry effluents have heavy metals, which are a threat to ecological balance. Hence, sensing and quantifying these ions are in high demand.

The analytical field has recently become more efficient in exploring the qualitative and quantitative information related to human survival. The techniques such as ion-selective electrodes, ion chromatography, voltammetry inductively coupled plasma mass spectroscopy, and fluorescent chemosensors have played a significant role in identifying the pollutants and metal ions [31–34]. These traditional methods have limitations because of costly instruments, strenuous procedures, need for expertise, etc. To fill in the drawbacks of the analytical techniques, photophysical properties such as absorption and fluorescence behavior of the compounds are studied, giving immense information about the compounds. Fluorescence is a luminescence that emits light energy previously absorbed, specifically from singlet excited state to ground state. As it is an emission spectrum, it gives additional information than absorption spectra. Thus, the fluorescence technique has its significance in chemosensing, which senses and quantifies metal ions. Fluorescent chemosensors have unique specifications because of their visual simplicity, sensitivity, selectivity, and rapid response. Hence it is mainly used to investigate the molecular process in which it acts as a molecular stopwatch, starting when the light is on and ends when the light is off. Designing such molecular systems for sensing metal ions and biomolecules through naked-eye detection is an exciting goal. Different sensing mechanisms take place during sensing, such as Fluorescence resonance energy transfer (FRET) [35], Intramolecular charge transfer (CT) [36], and Chelation enhanced fluorescence (CHEF) [37], Photoelectron transfer (PET) [38], Excimer formation [39], etc.

**Fig. 1:**
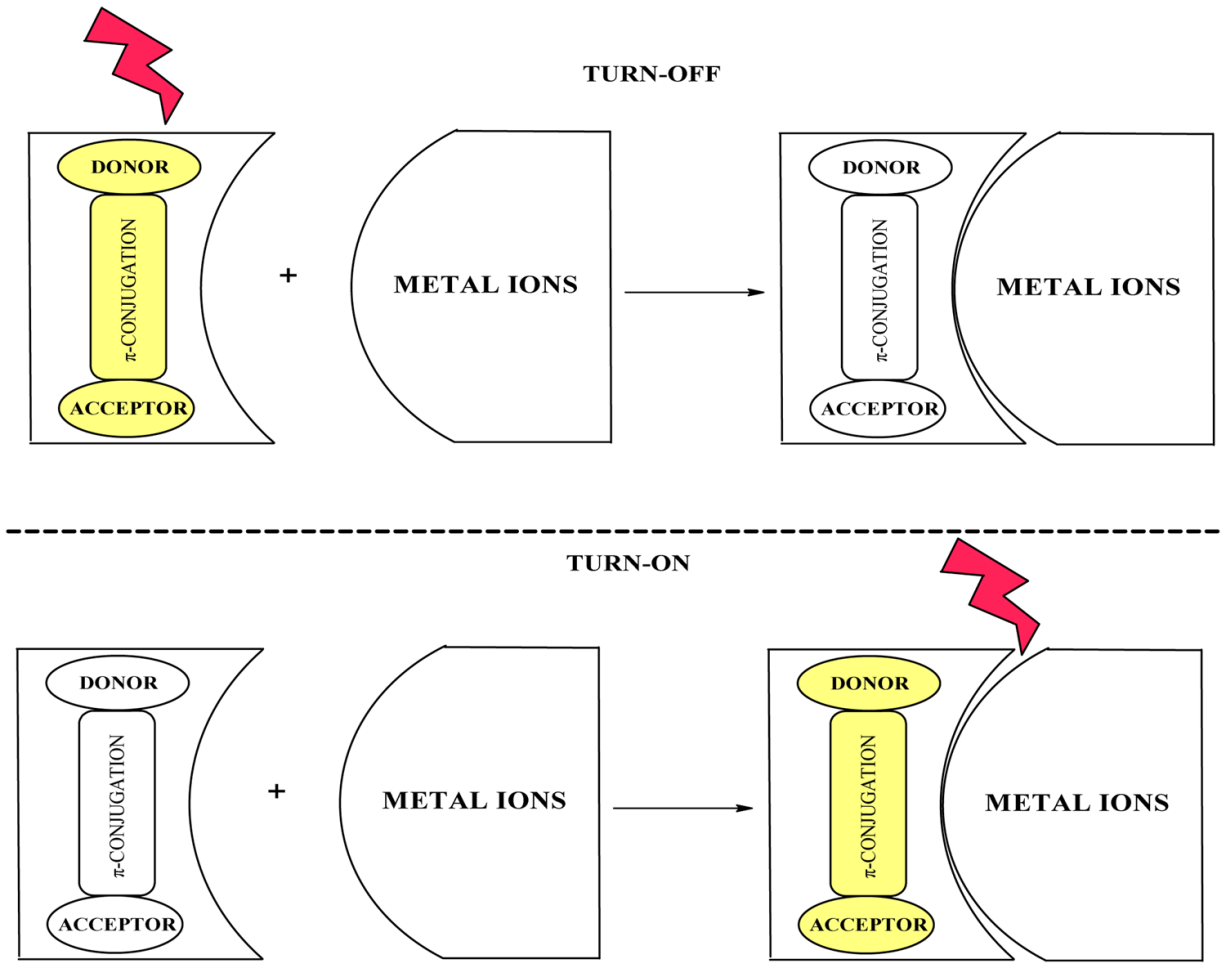
Schematic representation of fluorescent probes

There are numerous organic compounds known for their chemosensing properties, among which chalcone derivatives have their forte because of flexibility in structural modification. The chalcone derivative's selectivity and sensitivity can be modified by incorporating the aromatic groups with different electron donors and acceptor substituents. Chalcone derivatives with the appropriate structure are thought to be worthy for the formation of fluorescent compounds. Pyrazolines were often used as a chemosensor for metal ions because of their structural variability. Pyrazoline can acts as an acceptor when it is integrated with donor groups and separated by the π-conjugation. This type of arrangement facilitates the internal charge transfer of electrons results in good emission intensity. Besides pyrazoline, many other chalcone decorated compounds were also reported as sensors like porphyrininc-pyrazoline, ferrocene-pyrazoline, siloxy decorated chalcones, nanosensors anchored on mesoporous silicates, etc. The presence of multiple hetero atoms increases the possibility of the attachment of metal ions to the targeted compound. In addition to this, sensitivity and selectivity are altered by the attachment of different substituents in the targeted compound phenyl rings. There can be two ways of cation sensing in fluorescence, either by turn-off process or turn-on process. In a turn-off phenomenon, initial emission intensity was quenched during sensing; in contrast, intensity enhancement is observed in the turn-on process (Fig. 1).

Gupta and co-workers summerised the chalcone derivatives as chemosensors in 2020 [40]. The review focussed only on the true chalcone skeleton as sensors but in this review we tried to focus light on derivatives of chalcones as metal ion sensors from 2001 to 2020. Due to physical or chemical interaction between the probe and metal ions, emission and absorption properties were changed, which was measured, and it is considered to gauge the sensing ability of the designed probes. Here we have included all possible reported probes and divided into single ion and multi-ion sensor. The metal ions summed up here are Fe^3+^, Hg^2+^, Ni^2+^, Zn^2+^ and Cu^2+^, Pd^2+^, Sb^2+^, Co^2+^, Mn^2+^, Fe^2+^, Cd^2+^, and Al^3+^.The practical application of these reported probes was also discussed to explore real-world application in cation sensing.

## Single Ion Sensor

### Fe^3+^

Iron is the fourth most plentiful element on the earth; it covers about ≈ 5.6%. It plays a vital role in biological systems. Its deficiency or superfluity may lead to the malfunctioning of the body system. As it is necessitated in blood production, its inadequacy results in anemia. Excess iron also leads to diabetics, heart disease, and liver problems. So, there is a need to develop a simple, quick, and cost-effective chemosensor to detect iron.

**Fig. 2:**
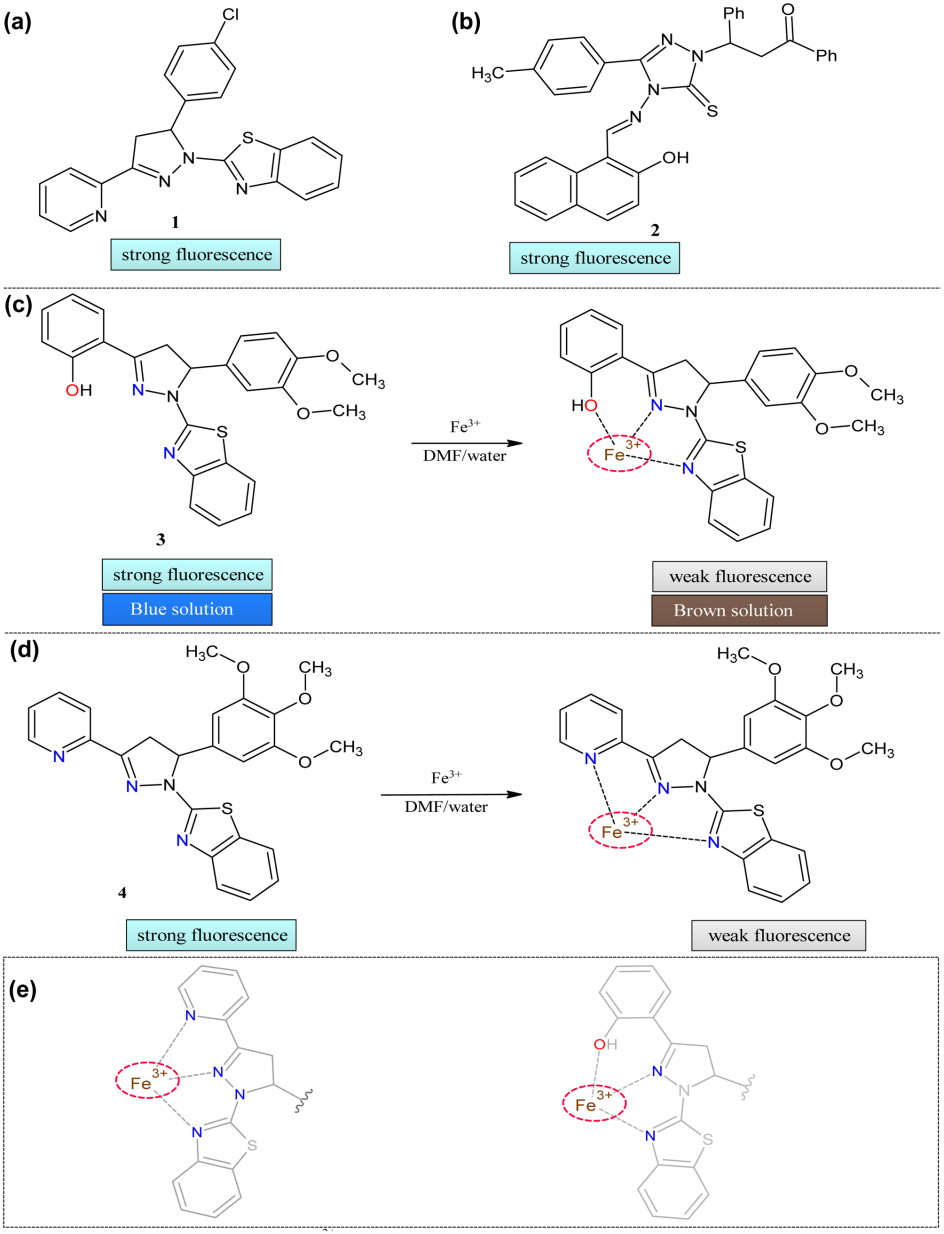
Pyrazoline based probes for Fe^3+^ ion sensing (**a**) **1** (**b**) **2** (**c**) **3** and (**d**) **4**. (**e**) Structural requirement for the Fe^3+^ ion sensing

The rigid and partly unsaturated moiety of 1,3,5-Triaryl-2-pyrazolines shows blue fluorescence [41, 42] with good quantum [43, 44]; Hu and his group reported the first pyrazoline-based chemosensor **1** for the detection of ferric ions, using the pyridine and benzothiazole group (Fig. 2a) [45]. The change in absorption and emission characteristics enables **1** to detect the ion in THF: water (95:5) medium. Upon adding Fe^3+^ ion, quantum yield decreases from 0.48 to 0.02 by forming a 1:1 complex with probe 1. The absorption spectra showed a hypsochromic shift from 354 to 337 nm with enhancement in absorption intensity. The change in intensity was because of an electron transfer from the pyridine, benzothiazole, and pyrazoline group of 1 to Fe^3+^ metal ion than any other ions under study. The fluorimetry showed a peak at 462 nm (ɸ = 0.54), which decreased with the addition of Fe^3+^ ions (ɸ = 0.02). The selectivity was tested over the other metals, but only the Fe^3+^ ion showed significant quenching. The Jobs plot indicated the formation of a 1:1 metal to ligand complex. The LOD was 1.4 µM. The quenching of intensity was observed because of the energy/electron transfer from the ligand to the metal.

Schiff bases are known for their excellent sensing ability towards the anions, alkaline earth metals, and heavy metals [46, 47]. These Schiff bases having triazolethione moiety as sensor **2** were used by Wang et al. (Fig. 2b) [48]. The sensing property was analyzed by absorption and emission techniques. The absorption maximum was found to be at 367 nm in the free state, and then upon the addition of metal ions, it gets blue-shifted to 358 nm with increased intensity. Spectrofluorimetric measurement showed intensity enhancement with the redshift of about 18 nm from 445 nm emission maxima. The Jobs plot showed that the metal to ligand complex ratio was 1:1.

Khan et al*.* followed a similar strategy and reported fluorescent chemosensor **3** with phenolic moiety instead of pyridine group as coordinating ligand (Fig. 2c) [49]. In the solvent DMF: water (9:1), sensor **2** showed good emission intensity(ɸ = 0.46) without metal ions. Upon addition of Fe^3+^ ions, a sevenfold decrease in fluorescence intensity(ɸ = 0.075) was observed with color change from greenish blue to brown. This quenching behavior was observed because of chelation-enhanced quenching phenomena. To gauge the selectivity, titration was performed in the presence of the other metal ions, which showed that the ligand selectively detects the Fe^3+^ ions. This selectivity is because of the good coordinating capacity of ferric ion to the pyridine and benzothiazole moiety of the sensor by chelation-enhanced quenching. The quenching behavior was confirmed by the Stern–Volmer plot with nonlinearity indicating the static or dynamic quenching.

Asiri et al*.* reported a similar fluorescent sensor **4** as 1 just by replacing the chlorobenzyl group with the trimethoxy benzyl group for the Fe^3+^ ion by fluorescence quenching mechanism in aqueous DMF (Fig. 2d) [50]. This replacement does not change the quantum yield much compared to sensor 1. The quantum yield decreased from 0.5 to 0.09 on the addition of the metal ions. The stoichiometry of the metal and chemosensor **4** was 1:1. The solvatochromic study showed that the emission spectrum was influenced more than the absorption spectra by the solvent. In a non-polar solvent, structured emission was observed, but in a polar solvent, structure-less emission was observed. The emission band at 364 nm was observed at excitation wavelength 355 nm. The intramolecular charge transfer was favored in **4** because of the presence of pyridine, pyrazoline, and benzothiazole moieties. The Fe^3+^ ion forms complex with pyrazoline moiety and, hence because of LMCT (ligand to metal charge transfer), fluorescence quenching was observed. Other metal ions were unable to form the complexes because of the geometry of the sensor and the ionic radius of the metal ions.

We can conclude from these findings that the presence of the benzothiazole group increases the possibility of recognition of the ferric ions. It is essential to figure out that Fe^3+^ ions form a five-membered ring with hetero atoms of the ligands involved. Even though the chemosensor 1 and 2 sensing mechanisms were not proposed, we can infer that the heteroatoms are in a suitable position to form the five-membered ring. So, if ligands possess electron-donating atoms in appropriate positions such that it forms five-membered rings with the incoming metal ions, then there is more probability of sensing these ferric ions (Fig. 2e). So, in the above cases, azole and benzothiazole played a versatile role in the site of binding and warranted the accurate geometry for the stability of the metal complex. Table 1 portrays the abilities of chalcone-based chemosensors for the detection of iron.

**Table 1:** Comparison of parameters of Fe^3+^ sensors

**Probe**	**Measured Signal**	**Stoichiometry**	**LOD**	**Quantum Yield of the probe**	**Real Samples**
**1**	UV–Vis/Fluorescence	1:1	1.4 µM	0.48	–
**2**	UV–Vis/Fluorescence	1:1	–	–	–
**3**	Fluorescence	–	0.1 µM	0.46	–
**4**	Fluorescence	1:1		0.5	–

### Cu^2+^

Copper is the third most copious trace element in human beings. According to WHO, the upper intake level of copper per day is 10 mg. The decrease or increase of the measure for long days may be detrimental to the biological systems. The lower amount may lead to health risks such as ataxia, anemia, etc. The copper toxicity may cause jaundice, melena, hypotension, etc. So, sensing and quantifying the metal ion is more important for chemical analysis and environmental protection.

**Fig. 3:**
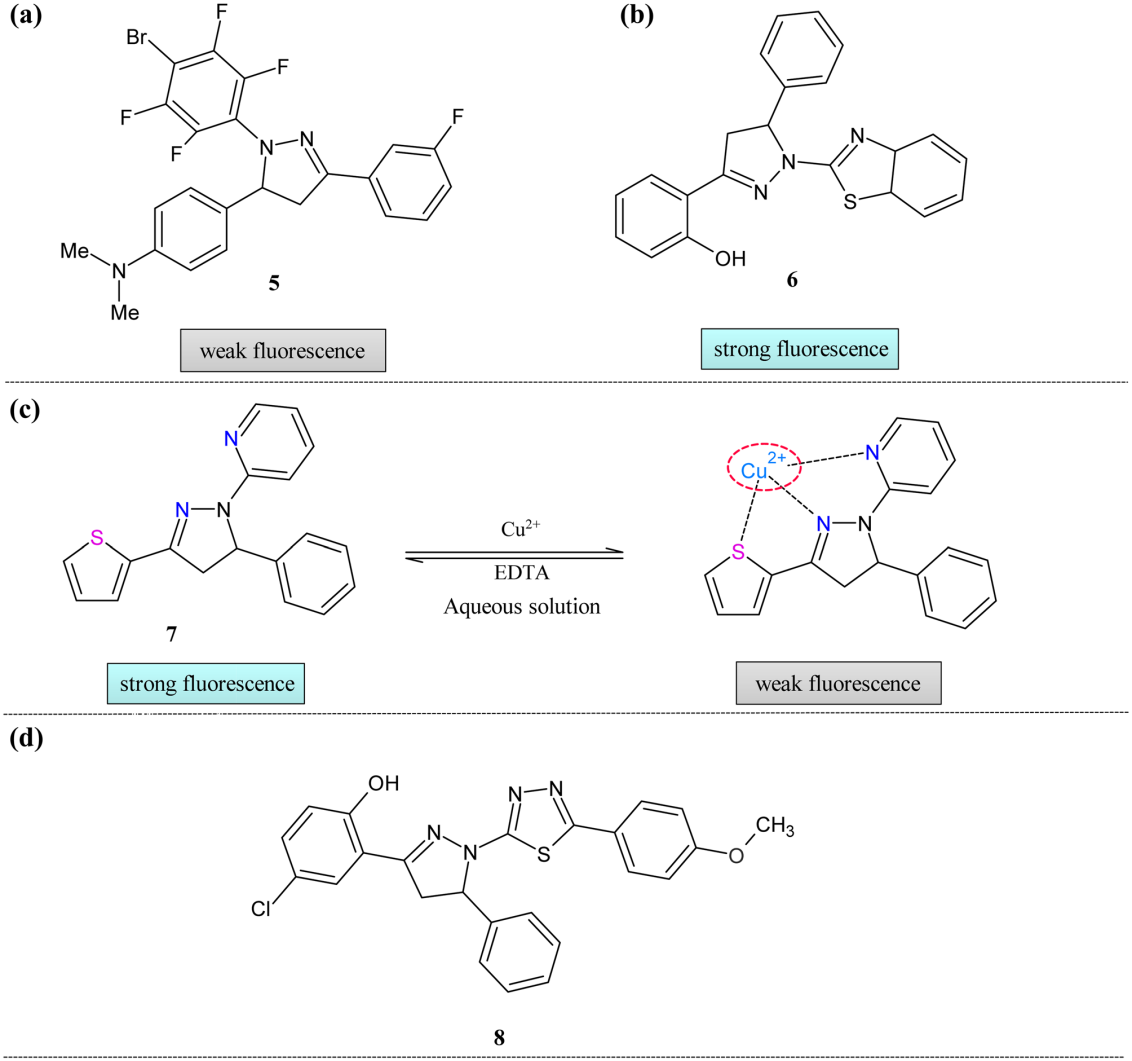
Pyrazoline based probes for Cu^2+^ ion sensing (**a**) **5** (**b**) **6** (**c**) **7** and (**d**) **8**

Encouraged by the need for the simple and reactive copper sensor for quick detection, Lu et al*.* reported the pyrazoline-based chemosensor **5** (Fig. 3a) [51]. This pyrazoline-based chemosensor detected the copper ion with an enhancement of fluorescence intensity in the solvent mixture ethanol: water (1:7) at pH = 7. The reaction mechanism was not explained fully but predicted that electrons might flow from nitrogen atom of pyrazoline to Cu^2+^ ions on coordination.

In 2012 Hu et al. reported the pyrazoline-based sensor **6** for the copper ions. The sensor showed a good quantum yield(ɸ = 0.2) in the beginning, but upon the addition of metal ions, it decreased (ɸ = 0.025) because of almost 12% fluorescence quenching [52] (Fig. 3b). The free ligand showed the absorption band at 352 nm. This band synchronously get decreases in the presence of metal ions with the new band at 400 nm. The fluorescence titration showed an emission maximum at 438 nm. The detection limit was found to be 8.7 × 10^–8^ M. This prepared ligand had tolerance over the other competing metal ions. Anionic counter-part effects were studied using other salts of the copper metal such as chloride, nitrate, and sulfate, which showed no significant changes in the intensity. The reversibility of the complex formation was studied by adding EDTA. The increment in the fluorescence intensity showed the free chemosensor's regeneration because of the complex formation between the EDTA and copper ions. The quenching behavior was observed because of an electron transfer from the phenol, benzothiazole, and pyrazoline moiety.

In the literature, there are many chemosensors for copper ions based on fluorescein [53], indole [54], quinoline [55], coumarin [56], etc. But these sensors had many disadvantages like reversibility [57], low selectivity [58], and solubility in water [59]. To overcome the shortcomings of the reported chemosensor, thiopheyl pyrazoline-based chemosensor **7** was reported by Li and the group [60] (Fig. 3c). The probe showed maximum absorption at 352 nm; it gets redshifted by about 10 nm upon adding the copper ion with the decrease in the absorption. The fluorescence study showed that the intensity peak at 452 nm was decreased only for copper ions. The limit of detection (LOD) was 1.919 × 10^−7^ M. The selectivity of the probe was tested in the presence of the other competing metal ions. As an extension of selectivity, the anion effect was studied in copper salts such as nitrates, chlorides, acetates, and sulfates. The stoichiometry of the metal to the sensor was 1:1. The reversibility of the complex was checked with EDTA, which indicates the regeneration of the chemosensor. The pH effect revealed that the complex was stable only at 7–7.5. The coordination mechanism was proved by density functional theory which indicates the possibility of coordination by pyrazoline, pyridine, and thiophene moieties. To study the selectivity in the living cell, HeLa cells were used. The study depicted that the cell image was initially highly fluorescent, but the intensity was fully quenched with the addition of copper ions.

The substituents influence the photophysical properties of the pyrazoline rings at the 1- and 3- positions [61]. Influenced by these facts, Wang et al. introduced the 1,3,4-thiadiazole group at 1- position and reported an excellent blue fluorescent compound as a copper ion sensor **8 **(Fig. 3d) [62]. The solvent effect on the photophysical properties revealed that absorption spectra did not vary much, but fluorescence intensity redshifted. In the fluorescence study, as they increased the concentration initially, the intensity was increased but after 5 × 10^−5^ M sudden decrease in intensity was observed because of the collision. The absorption spectra showed that the initial band at 375 nm was decreased in intensity and followed by the formation of new peaks in the range of 390-410 nm. The competition experiments are conducted to overcome the complex background of other competing ions. The only slight disturbance caused in intensity, suggesting good selectivity of the ligand over the other metal ions.

**Fig. 4:**
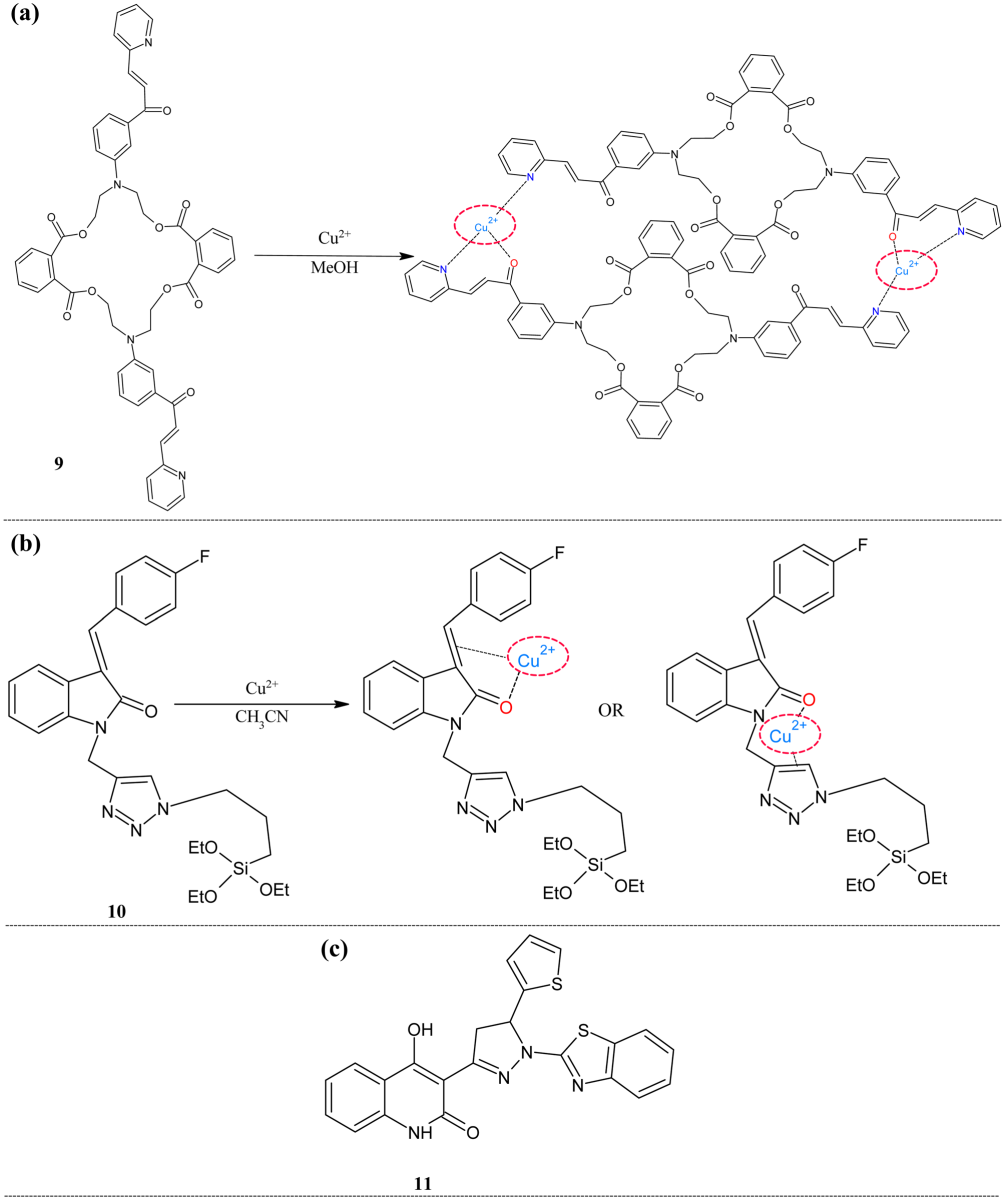
Probes for Cu^2+^ ion sensing (**a**) Macrolides-chalcone framework **9** (**b**)Triazole based **10** and (**c**) pyrazoline based **11**

In recent years there has been more focus on incorporating the biologically active groups into the chalcone to scrutinize the novel activities. In such one attempt, Yang et al. introduced the macrolides into the chalcone framework and analyzed the metal ion sensing ability [63] (Fig. 4a). These macrolides structurally have more than 12 rings with ester bonds which portray a wide range of biological properties. The free sensor **9** showed two absorption peaks at 261 and 303 nm. There was no change in these peaks on the addition of metal ions except copper. The presence of copper causes the disappearance of peaks at 303 nm and new peaks at 339 nm. It was negotiated that there was a possibility of transferring electrons from the nitrogen of the pyridine moiety.

The 1,3-dipolar reaction may produce a highly conjugated system with diverse functionalization. To bring knowledge into practical application siloxy based isatin chalcones were used by Singh et al*.* to form a flexible conjugate plane with the incoming analyte [64] (Fig. 4b). This analyte was an indolin-2-one based scaffold of chalcone functionalized with a siloxy framework. The targeted ligand was synthesized by the green method as microwave-assisted synthesis. The UV–Visible study showed that the free analyte **10** exhibits peaks at 260 and 340 nm in CH_3_CN; these peaks are unchanged even with all other competing transition metal ions, but in the presence of copper ions got shifted to 460 and 315 nm. This shift occurs because the complex formation through the oxygen and double bond of chalcone moiety result in increased conjugation, which leads to bathochromic shifts. The limit of detection was 3.21 µM which is found below the WHO assigned value (20 µM). The binding stoichiometry of the complex was 1:1. Two different modes of the mechanism were proposed for the sensing. In one mode, oxygen and chalcone double bonds were involved, and in another, triazole nitrogen and oxygen of carbonyl were presented. The HSAB concept supported this binding mode because copper being borderline acid it prefers to form a complex with a borderline base like an oxygen atom.

As an extension of sensing work, Subashini et al. reported quinoline-pyrazoline scaffold as a dual sensor for copper and sulfide ions [65] (Fig. 4c). Sensor **11** exhibited an excellent absorption peak at 377 nm in water: DMSO (9:1) in phosphate-buffered saline (pH = 7). The synchronous decrease in the maximum peak was observed with a new peak appeared at 386 nm for the copper ions and with no distinct change for other cations. A fluorescence study was conducted to scrutinize selectivity, which unveiled that **11** showed emission maximum was at 463 nm (ɸ = 0.4398). This intensity was quenched on adding copper ions (ɸ = 0.02) with color change from strong blue fluorescence to colorless under UV irradiation. The effect pH was revealed that this quenching was observed throughout the pH range from 0–12. The competition experiments showed that only Mn^2+^ and Ni^2+^ muddled the intensity of **11**. From the study, it was noted that stoichiometry was 1:1, and LOD was 0.16 nM. The fluorescence response of this complex to the sulfide ion was studied since copper can form very stable species CuS. This practical knowledge was also proved here, on the addition of S^2−^ ions fluorescence intensity increased at 459 nm, which was similar to the original free analyte. The addition of other anions as CN^−^, Cl^−^, Br^−^, I^−^, NO_2_^−^, NO_3_^−^ and AcO^−^ caused slight changes. In addition, sulfide having amino acids (GSH,1-Cys,1-Hcys) were also studied. But the complex was selective only for the S^2−^. The detection limit was found to be 0.2 µM. The formation of the metal complex was confirmed by the DFT study, which revealed that the molecule's stability increased after the complex formation. The positions of the HOMO and LUMO also confirmed the formation of bond between the metal and ligand. The practical applicability of the analyte was proved by cell imaging in MG63 cells. The IC50 value was 20.67 µM, indicating the non-toxicity behavior of sensor **11**. The fluorescence image in cells proved the on–off-on behavior, the intensity was quenched on the addition of copper ions, but it was regenerated upon the addition of the sulfide ions. The potential sensor application for S^2−^ ions detection in effluent water studied the results portray that amount was similar to the iodometry.

**Fig. 5:**
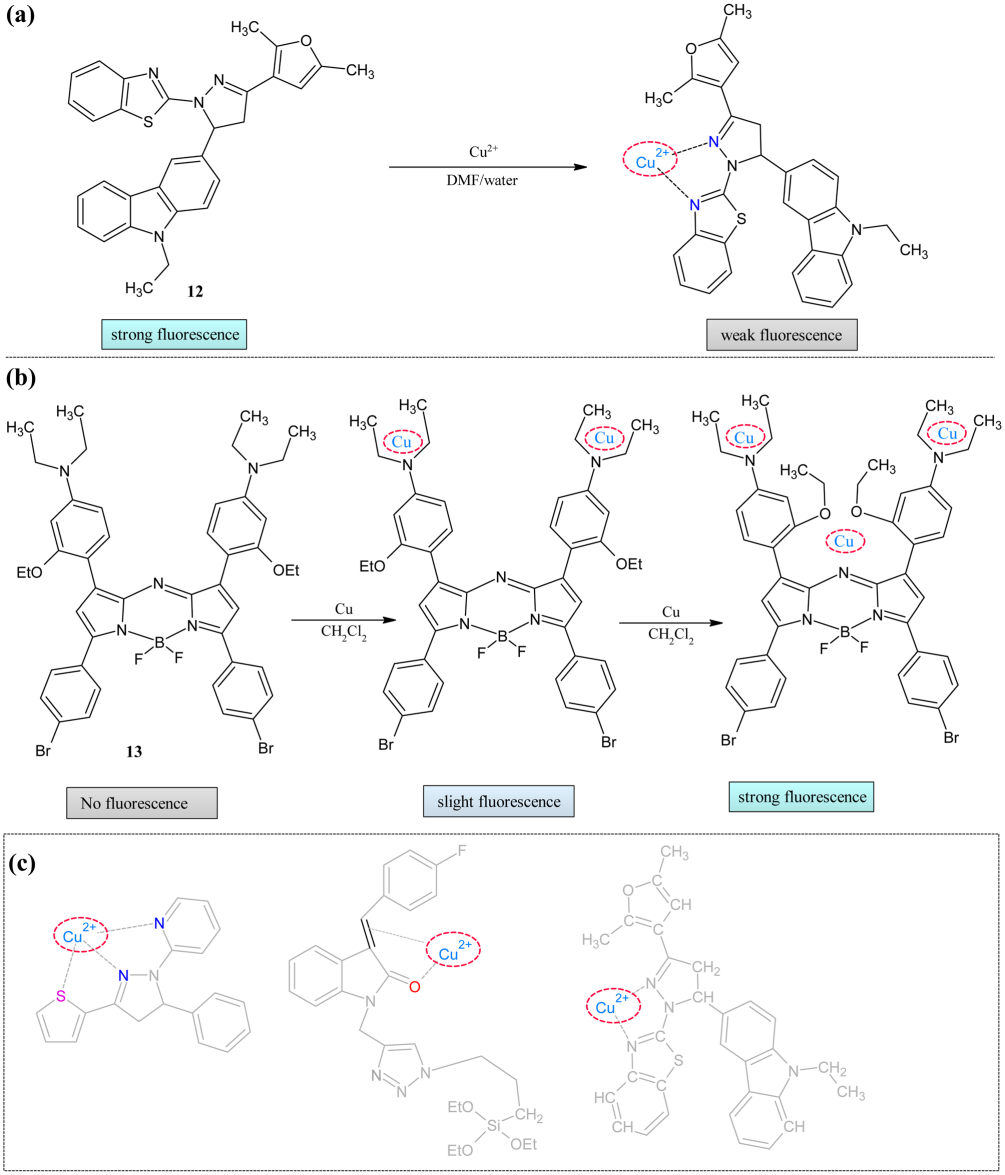
Cu^2+^ ion sensors (**a**) Hydrazinobenzothiazole framework **12** (**b**)Aza-BODIPY framework **13** and (**c**) Five-membered structural requirement for sensing

The heterocyclic systems with donor-π-acceptor moiety usually show good blue fluorescence because of intramolecular charge transfer (ICT) [66]. Pyrazoline is the one among these which acts as an acceptor; when it is joined with donor groups, continuous delocalization of π-electrons takes place, which displays excellent blue fluorescence [67]. One such representative example is the study by A. Khan and Praveen Kumar, in which they equipped carbazole and hydrazinobenzothiazole with the pyrazoline moiety **12** (Fig. 5a) [66]. The photophysical properties were studied in different solvents, from polar to non-polar. In the absorption study, when the polarity was increased, the hyperchromic shift was observed, revealing that the ground state of sensor **12** has a more polar nature. The fluorescence spectrum displayed bathochromic shifts in emission maxima of about 28 nm from n-heptane to the DMSO. This value was more than the absorption shift indicating that the excited energy state was more affected than the ground state. Initially, sensor **12** showed a good blue fluorescence (ɸ = 0.68) at 420 nm in DMF: water (9:1) which was quenched 7 times (ɸ = 0.2) on the addition of the copper metal ions with color changing to colorless. The quenching was observed because of the chelation-enhanced quenching. The quenching was observed because of the transfer of an electron from pyrazoline and benzothiazole group to a vacant d orbital of Cu^2+^ ion. The Hildebrand and Stern–Volmer plot also explained the quenching behavior. The cationic and anionic surfactant's effect on the analyte was also examined in the presence of the CTAB and SDS. They observed that redshift with increasing concentration of the surfactants because the micelle domain polarity influenced the ICT states.

The main disadvantages of the previously reported copper sensors are they absorb the radiation in the UV–visible region, which is the same for the fluorescence spectra. So, there was a need to develop near-infrared (NIR) based probes for these ions. The aza-BODIPYs shows excellent photophysical properties in the NIR region [68, 69]. Influenced by these facts, Gawale et al. reported the BODIPY based sensors **13** for the copper ions [70] (Fig. 5b). The solvatochromic study showed three absorption peaks for polar solvents, but for non-polar solvents, two peaks were observed. When solvent polarity increases, there was a redshift observed. The probe's sensitivity towards the various mono and divalent ions was analyzed, which portrays that the probe exhibits absorption changes at 710 nm only for copper ions, with color changing from blue to purple. To understand the binding events, various equivalents of the copper ions were titrated, which showed slight changes in the intensity of the peak with three isosbestic points at 742 nm,600 nm, and 685 nm indicating varying structures of metal and ligand. Further increase (above 2 equivalents) decreases the peak at 742 nm and increases at 450 nm. In the fluorescence study, the binding of the probe with copper ions at two equivalents showed two peaks at 620 and 700 nm. This peak at 700 nm further increased upon adding 3 equivalents of metal ions; the trend was followed until 8 equivalents; after that, it got saturated. The LOD for the probe was low(350 nm). The binding capacity of the probe was enhanced because of the lone pairs of electrons on nitrogen; at lower equivalents, peripheral nitrogen accommodated the incoming copper ions, which was proved by the blue shift in the spectra, on further increased metal ions were attracted by the core nitrogen and ethoxy moiety. The fluorescence enhancement was observed because of the restricted rotation of phenyl moiety on coordination with metal ion. The FTIR and NMR supported this binding mechanism with redshift in C-O(OCH_3_) and C = N stretch and the downfield peak of OCH_3_ and N(CH_3_)_2,_ respectively.

From the above literature analysis, copper can indeed form five-membered stable complexes, especially with the pyrazoline rings. In **9** and **13,** it formed seven-membered rings indicating the further possibility (Fig. 5c). In many other cases, even though the mechanism was not explained, but we can conclude from the position of the heteroatoms, there may be a chance for the formation of the five-membered rings except in **5**. Probe **11** showed the lowest possible LOD among the listed other sensor with good quantum yield. The variance of sensor behavior is shown in Table 2.

**Table 2:** Comparison of parameters of Cu^2+^ sensors

**Probe**	**Measured Signal**	**Stoichiometry**	**LOD**	**Quantum Yield of the probe**	**Real Samples**
**5**	Fluorescence	–	–	–	–
**6**	UV–Vis/Fluorescence	–	8.7 × 10^–8^ M	0.2	–
**7**	UV–Vis/Fluorescence	1:1	1.91 × 10^–8^ M	–	–
**8**	Fluorescence	–	–	–	–
**9**	UV–Visible	–	–	–	–
**10**	UV–Visible	1:1	3.21 µM	–	–
**11**	UV–Vis/Fluorescence	1:1	0.16 nM	0.43	MG63 cell imaging
**12**	UV–Vis/Fluorescence	–	–	0.68	
**13**	UV–Vis/Fluorescence	–	350 nM	0.02	–

### Hg^2+^

Mercury was the most dangerous global pollutant because of its trouble-free solubility in water. In addition to this, it can pass through the skin quickly, leading to damage to the body's internal parts. Even a minute quantity of this metal may cause central nervous system damage. It may also lead to serious health issues like genotoxic, Hunter-Russell syndrome, cognitive disorder, Minimata, Alzheimer's disease, etc. [71, 72]. The primary sources are the ore industry, oil refining, rubber processing, pollution from power plants, and solid waste incineration [73]. The maximum level of Hg^2+^ metal in drinking water by WHO was 2µgL^−1^ [74].

**Fig. 6:**
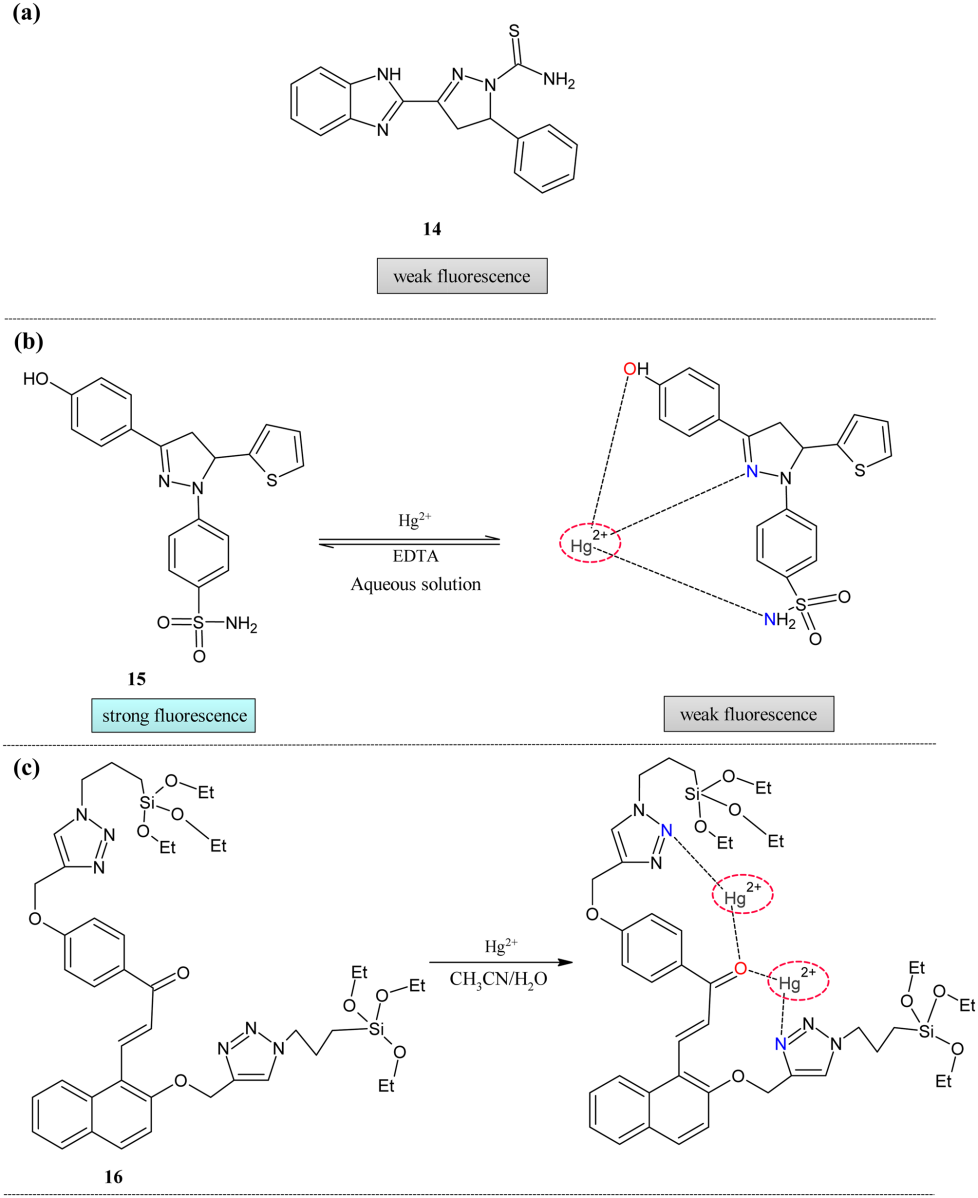
Hg^2+^ ion sensors (**a**)Pyrazoline framework **14** (**b**)Benzenesulfonamide decorated pyrazoline framework **15** and (**c**) Siloxy decorated Triazole group **16**

Even though many chemosensors were reported in the literature based on biomolecules, nanoparticles, and polymers, there was still a need to develop a simple and quick sensor as these sensors suffer drawbacks like poor selectivity of metals [75] and delayed response [76]. Influenced by these factors, Wang et al. developed pyrazoline-based chemosensor **14 **(Fig. 6a) [77]. In 10% ethanol solution, the probe showed an absorption band at 347 nm. The hypsochromic shift of absorption band was observed for metal ions Cu^2+^, Hg^2+^, and Ag^+^ at 317, 292, and 319 nm. The titration experiment with Hg^2+^ showed decreased absorption at 344 nm and a new band with increased intensity. The isosbestic points at 328 nm indicate the equilibrium between the metal and ligand. The selectivity of the probe towards the metal ions was tested by the fluorescence study. The intensity enhancement was observed with the addition of Hg^2+^ of about 26-fold. This increase was observed even with the Cu^2+^ and Ag^+^ of about five and eight-fold. The Jobs plot showed that the stoichiometric ratio was 1:1. The quantum yield of the free probe was 0.0465; this was increased to 0.859 for the complexes. The LOD was found to be 3.85 × 10^–10^ M. The pH effect showed that the probe was suitable for detection in the pH range of 7 to 11. The NMR spectra proved the formation of the complex indicating the downfield of pyrazoline ring protons. The HeLa cell imaging of the probe showed a significant enhancement in the fluorescence intensity after adding the mercury ions. This provides visual proof for the sensing ability of the probe.

Compared with the different analytical methods, chemosensors play a vital role in the sensing field because of rapid response, sensitivity, and selectivity towards the targeted analyte. To improve the sensing behavior of previously reported pyrazolines, E.Bozkurt and H. I Gul reported probe **15** with few improvements in the basic structure of the pyrazoline [78] (Fig. 6b). The pyrazoline moiety was decorated with the benzenesulfonamide group to enhance the sensing ability. The absorption titration revealed no effect on the absorption of the probe in the presence of the various metal ions. But Fluorescence measurement showed decreased intensity at 449 nm only for mercury ions. The photographs of the complex solution color change for Hg^2+^ under UV-light. The quantum yield was reduced from 0.68 to 0.16. The fluorescence lifetime of the compound was the same for all the metal complexes except mercury, for which it was decreased. The LOD of the compound was 14.54 µM. The interaction ratio of the complex was 1:1. To check the reusability of the probe, EDTA was added, resulting in the regeneration of the emission intensity. These changes were repeated for three cycles indicating sensitivity was not disturbed for the probe. In the presence of other interfering ions, the selectivity of the probe was not disturbed. Sensor **15** showed a rapid response (1 min) to the metal ions with prolonged continuity of the complex nature of about 15 min. The pH effect showed the ON–OFF-ON behavior for the free and complexed probe in the range of pH 2–12. Below 10, there was high intensity while at 10 intensity decreased then increase in intensity was observed at 12. The lifetime measurements also confirmed the formation of the complex. The lifetime of **15** in pure water in the absence and the presence of metal ions was the same for all metal ions except for Hg^2+^. The sensing mechanism was proved by the IR, NMR (^1^H and ^13^C) spectral analysis. There was an interaction between the metal ion and hydroxy group of phenol, pyrazoline, and sulfonamide moieties. The practical application in a tap water sample with additionally added Hg^2+^ concentration was precisely detected.

To move into the next step in mercury ion detection, Singh and his group analyzed silicon-based molecular systems in the semi-aqueous medium [79]. The introduction of triazole group via click reaction set forth more than one coordinating site to the receptor **16 **(Fig. 6c). In the photophysical study, the absorption spectra revealed that Hg^2+^ decreased the absorption intensity because of the similar cage size and Hg^2+^ ion size. The spectra were recorded in CH_3_CN: H_2_O (9: 1). Upon adding the Hg^2+^ ions, systematic absorption changes were observed with blue shift and broadening the peaks. According to the HSAB principle, the selectivity of the probe was because of the stronger affinity of Hg^2+^ towards the N and O atoms of the ligand. The flexible structure of silane with hetero atoms oxygen and nitrogen facilitate the formation of the complex. The Jobs plot proved the formation of a.

The literature reveals no particular way that mercury can form the stable complex with the ligand. But it may be true that it can create a complex with the bi and tridentate ligand. The deviation of sensor property was tabulated in Table 3.

**Table 3:** Comparison of parameters of Hg^2+^ sensors

**Probe**	**Measured Signal**	**Stoichiometry**	**LOD**	**Quantum Yield of the probe**	**Real Samples**
**14**	UV–Vis/Fluorescence	1:1	3.85 × 10^–10^ M	0.0465	HeLa cell imaging
**15**	UV–Vis/Fluorescence	1:1	14.54 µM	0.68	Tap water
**16**	UV–Visible	1:2	0.1 µM	–	–

## Ni^2+^

Nickel was one of the essential trace elements in the body involved in many biological reactions [80]. It is also the heart of modern metallurgy involved in many processes such as electroplating, Ni–Cd batteries production, and alloy production [81]. The overuse of nickel products can lead to environmental pollution, which leads to nickel toxicity. This toxicity may cause kidney and heart-related problems, asthma and lung disorder, and cancers. Hence the monitoring of the nickel ions becomes crucial [82, 83].

**Fig. 7:**
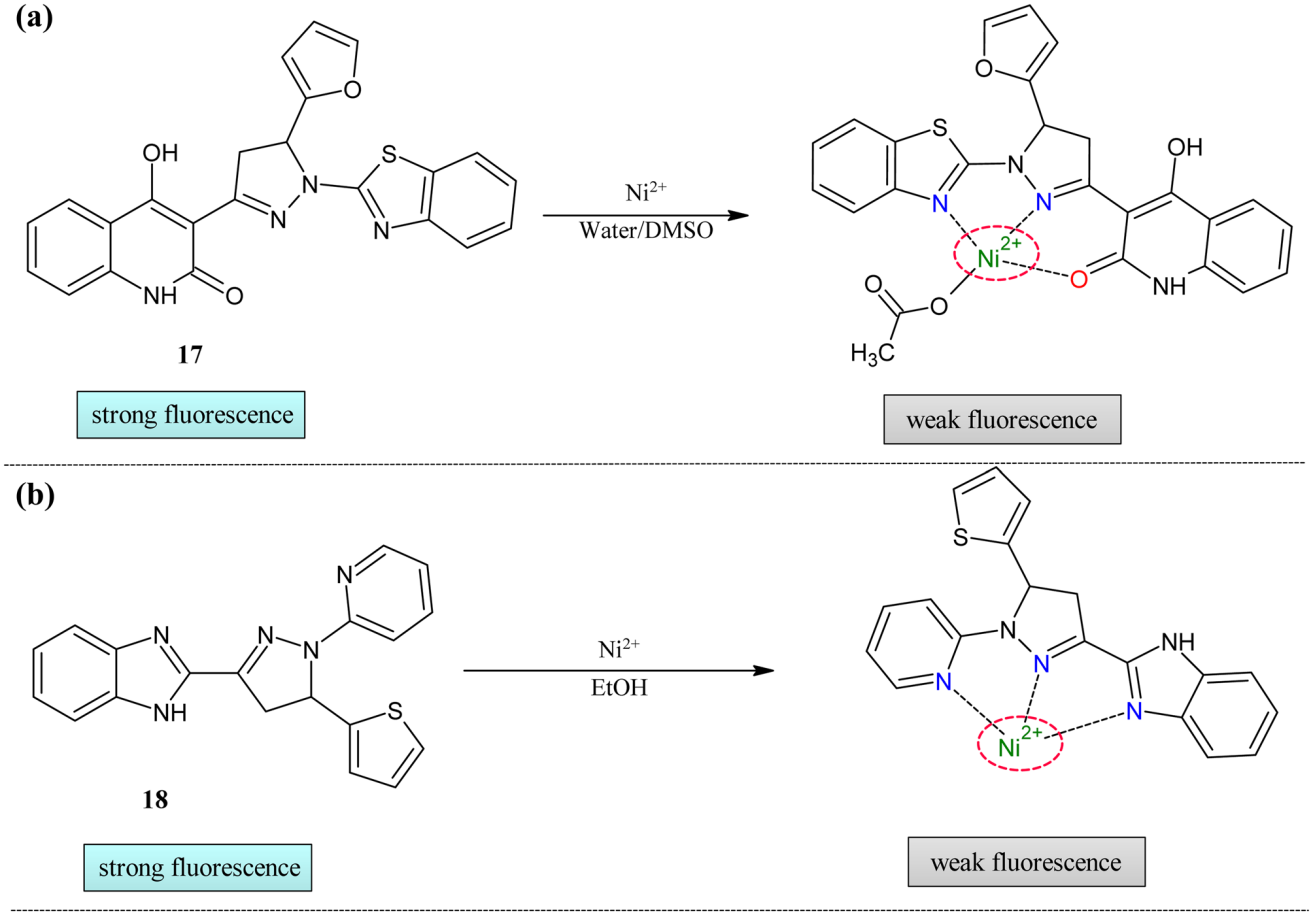
Ni^2+^ ion sensors (**a**)Pyrazoline framework **17** (**b**)Benzimidazole decorated pyrazoline framework **18**

Influenced by the excellent fluorescent properties of pyrazoline moiety, Subashini et al. reported the first pyrazoline-based probe **17** for nickel ion detection (Fig. 7a) [84]. The absorption and emission study were conducted in the water/DMSO (9:1). The absorption band was centered at 376 nm, and it was redshifted to 389 nm on the addition of the nickel ions. Due to the intramolecular charge transfer (ICT) probe, **17** showed a peak at 465 nm in emission spectra, and this was decreased with the addition of the Ni^2+^ ions. The quantum yield of the probe was reduced from 0.2695 to 0.015(18% decrease). The Job's plot showed the 0.5 molar fractions indicating the 1:1 formation of the complex between probe and meatal. The LOD from the calculation was found to be 5.48 × 10^−7^ M. The effect pH was studied at different pH, which showed that the probe was suitable for biological applications. The practical utility of the probe was tested in MG63 live-cell imaging. The probe was non-toxic under the experimental condition (IC_50_ = 30.99 µM). Using a fluorescence microscope, these cells were imaged, which showed good fluorescence at 30 °C within half an hour. After supplementation of 10 µM of NiCl_2_ within 10-min quenching was observed. The scanning electron micrographs was further proved the formation of the complex.

Encouraged by the suitable fluorescent property and rapid coordinating capacity of the benzimidazole group, Han et al. prepared benzimidazole incorporated pyrazoline sensor **18** for sensing Ni^2+^ ions (Fig. 7b) [85]. The absorption spectra of this with various di and trivalent metal ions changed in a very different manner. In particular, for Ni^2+^, the absorption peak at 362 nm decreased with the appearance of a new absorption peak at 307 nm. The fluorescence in ethanol showed the quenching in the emission intensity on the addition of Ni^2+^ ions with the decrease in quantum yield from 0.26 to 0.011. The stoichiometry of the complex was 1:1 proved by the Job's plot. To gauge the tolerance of the probe sensing was checked in the presence of the various other ions, emission was quenched in all cases but not to the extent of nickel ions. The anionic effect showed no noticeable change in intensity.

The nickel ion sensors were not explored much in the field of chalcone-based pyrazoline. So, there is more chance for exploring the chalcone-derived nickel sensors. The tabulated parameters of the reported probes are tabulated in Table 4.

**Table 4:** Comparison of parameters of Ni^2+^ sensors

**Probe**	**Measured Signal**	**Stoichiometry**	**LOD**	**Quantum Yield of the probe**	**Real Samples**
**17**	UV–Vis/Fluorescence	1:1	5.48 × 10^−7^ M	0.269	MG63 cell imaging
**18**	UV–Vis/Fluorescence	1:1	3.85 × 10^–10^ M	0.26	–

### Zn^2+^

After the iron, zinc was the second most abundant element in the body. It is involved in many vital biological functions, acting as an essential co-factor such as apoptosis, neural transmitter, gene expression regulator, mammalian reproduction, etc., [86–88]. According to the National Institute of Health allowed upper limit of zinc for adults is 40 mg/day. The varied intake of the metals may lead to the hampering of the biological systems leads to infantile diarrhea, Alzheimer's disease, ischemic stroke, and epilepsy [89–91]. It is more bewitching to make visible zinc in living cells [92, 93]. Because of this metal's diamagnetic properties and color-lessness, its role in most biological reactions is unanswered. In addition, selective detection of zinc becomes crucial because of the cadmium and zinc's similar electronic and binding properties.

**Fig. 8:**
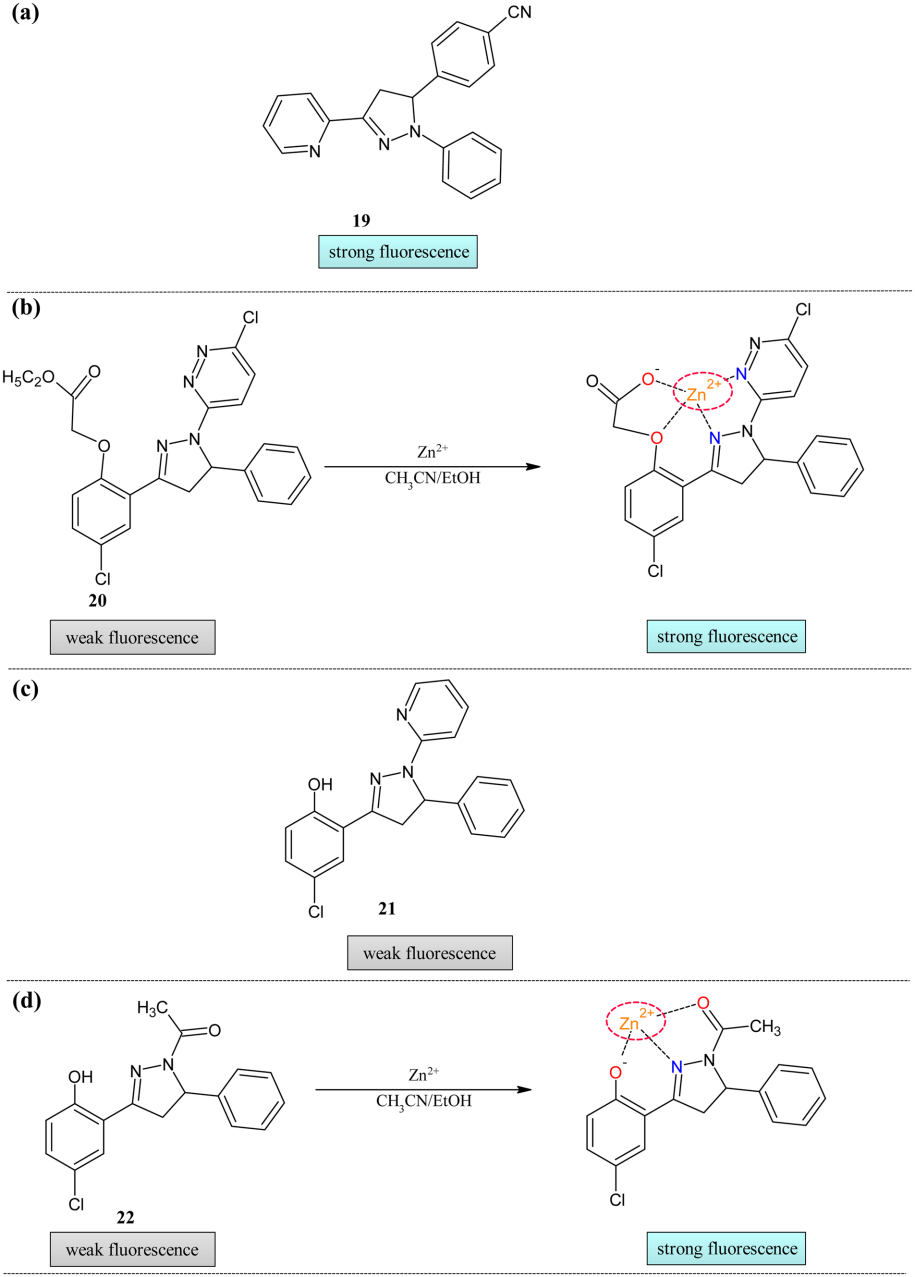
Zn^2+^ ion sensors (**a**) Pyridyl pyrazoline framework **19** (**b**) Ethyl acetate decorated pyrazoline moiety **20** (**c**) Pyridyl pyrazoline framework **21** (**d**) Acetyl pyrazoline probe **22**

The first zinc ion sensor was reported by Wang and his group, which was processing the pyridyl-pyrazoline moiety [94](Fig. 8a). In acetonitrile, this sensor **19** was more responsive towards transition metals and showed no interaction with alkali and alkaline earth metals. The transition metals such as Cu^2+^, Zn^2+^, Co^2+^, and Ni^2+^ showed decreased optical density at 360 nm in absorption spectra with broadening the peak due to π-π* transition. Fluorescence spectra showed decreased emission at 479 nm only for zinc ions with the appearance of a new band at 568 nm assigning the complex formation. The quantum yield has been reduced on the addition of the zinc ions from 0.057 to 0.037. The effect of alkali and alkaline earth metals was tested on the testing ability of the probe, but it showed good selectivity of the probe only towards the Zn^2+^ ions.

The pyrazoline compound's absorption properties were influenced by the phenyl ring rotation and medium of solvent [95, 96]. These may also affect the fluorescence behavior of the molecule. Influenced by the properties of the pyrazoline compounds, the ethyl acetate decorated turn-on fluorescent sensor **20** was reported by Gong et al. to identify zinc ions [97] (Fig. 8b). The maximum absorbance of probe **20** at 347 nm was decreased with the redshift of the other peaks (347 to 316 nm). Several isosbestic peaks proved the complex formation at 370, 256, 318, and 282 nm. In the emission study, **20** showed a very weak peak at 480 nm, which was increased sixfold on the addition of the zinc. This enhancement was caused by the chelation-enhanced fluorescence (CHEF) between the zinc and probe. There was a 1:1 ratio between the metal and complex. Anionic effects study revealed that other salts (NO^3−^, CH_3_COO^−^ and Cl^−^) could also induce similar changes. The LOD of the compound was 4 × 10^–7^ M.

To overcome the drawbacks (poor selectivity, low solubility, and slow response) of the reported zinc sensors based on the moieties such as coumarin [98], triazole [99], benzoxazole [100], indole [101], xanthene [102], etc., Gong et al. reported sensor **21** with slight modifications in the previous probe [103] (Fig. 8c). The main advantage of the sensor was applicability in an aqueous solution. The UV–Visible measurement showed that the free probe peak at 347 nm was decreased gradually on increasing the concentration of zinc metal, with the appearance of a new peak in the range of 400-420 nm. The isosbestic points were observed at 378 nm. The coordination stoichiometry was found to be 1:1. The quantum yield was measured by the emission spectra, which were found to be 0.12 for **21**. On titration of the probe solution with the zinc solution, intensity enhancement was observed(ɸ = 0.53) at 460 nm. The non-linear curve fitting graph showed 1:1 stoichiometry. The competition experiments showed that only zinc metals induce changes in the fluorescence intensity. The pH effect depicted that the emission intensity was increased till 7; further increase may decrease the intensity. The limit of detection for the quantitative study was found to be 0.12 µM. In exploring the anionic counter-part effects study was extended with chloride acetates and nitrates of zinc which showed no apparent changes in the emission.

**Fig. 9:**
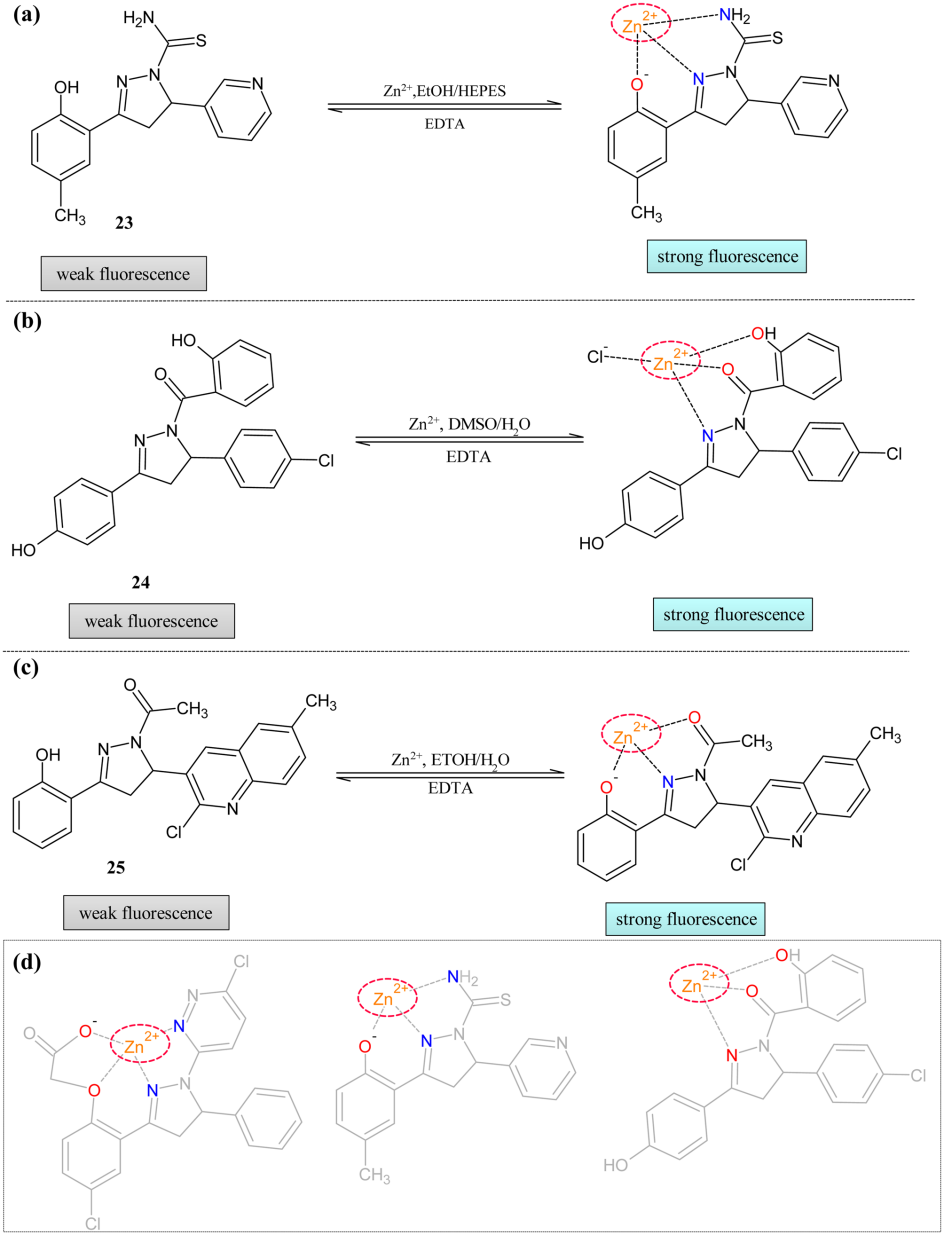
Pyrazoline-based Zn^2+^ ion sensor decorated with (**a**) Carbothioamide framework **23** (**b**)Triaryl framework **24** (**c**)Quinoline framework **25** (**d**) Five and six-membered structural requirement

By considering the practical applicability of the sensor, Zhang et al. synthesized sensor **22** for the zinc ion detection in neuron cells [104] (Fig. 8d). The UV–visible titration experiments showed the peak at 330 nm, which was decreased upon addition of the transition metal ions such as iron, cobalt, nickel, zinc, and copper with new band formation in the range of 360-425 nm. In fluorescence titration, only zinc ions showed the enhancement of the intensity at 468 nm. This is because the zinc's closed shell orbital avoids the fast non-radiative decay of the sensor. The tolerance of the Zn-**22** for biological applications was tested in the presence of the other alkali and transition metals. The intensity was stable for all tested ions except for the copper. The copper showed complete quenching of the intensity of this complex due stronger affinity of the probe towards the Cu^2+^. The stoichiometric ratio was 1:1. The quantum yield was increased from 0.023 to 0.25. The LOD of the probe was found to be 8.3 × 10^–7^ M. The probes could be regenerated by adding the EDTA ligand and further adding zinc again increase the intensity revealing the repeating usage of the probe for practical applicability. The effect of pH showed that both the Zn-**22** and probe were stable at physiological pH, but the complex intensity was decreased at higher pH. In neuron cells, SH-SY5Y intracellular zinc ion detection was performed using fluorescence microscopy. The cells exhibited very light fluorescence in the presence of the probe, but on the addition of the exogenous Zn^2+^, the fluorescence was visible, indicating the practical applicability of the prepared probe. The ^1^H NMR confirmed that phenolic OH group, pyrazoline, and methoxy group was involved in the bonding,

Li et al. reported the carbothioamide decorated pyrazoline moiety **23** for zinc detection [105] (Fig. 9a). This sensor showed the 80-fold turn-on response with good reversibility in lie cell imaging. In absorption spectra, zinc ions showed increased absorption at 338 nm with visible color change from colorless to yellow. At 390 nm new absorption band appeared with one isobestic point appeared at 364 nm. In addition to this, Cu^2+^, Co^2+^, and Ni^2+^ also showed increased absorption. The fluorescence response of the probe indicated the 80-fold increase in the intensity at 480 nm while the addition of the 10 equivalents of the zinc solution. This response was more superior compared to the previously reported zinc chemosensor. The competition experiments showed that selectivity was unaffected in the presence of the other metal ions. The quantum yield was changed from 0.0028 to 0.23. The Jobs plot showed the 1:1 bonding ratio between the metal and ligand. The probe could be used at physiological pH. The reversibility was of the probe was checked with the one equivalent of the EDTA, indicating complete quenching of the Zinc-**23** complex; further addition of the zinc solution enhanced the intensity proving the reversible binding of the metal and **23**. The involvement of the hydroxy group, pyrazoline, and -NH_2_ group was confirmed by the NMR and DFT calculations. The practical applicability was tested in the SH-SY5Y neuron cells. The fluorescence microscopic image showed that faint fluorescence, but on external addition of the zinc, fluorescence became visible, proving the potential biological application of the probe.

The triaryl pyrazoline-based turn-on sensor **24** was reported by Jeyanthi et al*.* [106]. The absorption study revealed that the peak at 308 nm was decreased with the appearance of a new peak at 372 nm (Fig. 9b). The isobestic point was at 350 nm. The fluorescence spectra of **24** in the presence of the various other metal ions were conducted in DMSO/H_2_O (8:2) at pH 7.4. The weak emission band (ɸ = 0.03) of **24** at 446 nm was enhanced (ɸ = 0.31) with the red shift to 458 nm. The Jobs method study and Electrospray ionization mass spectra (ESI–MS) showed the 1:1 equivalent formation complex. The LOD(1.02 µM) of the probe proved to be **24** was a more effective sensor for zinc detection. The presence of the other metal ions showed an insignificant effect on the functioning of the probe. The NMR spectroscopy proved the binding interactions.

The quinolines are well-known for their excellent fluorescent property with good quantum yield, structural variability in the coordinating sites, and solid binding ability [107]. When pyrazoline and quinoline scaffolds are combined, there is a chance for enhancement in the photophysical behavior of the molecule. This combination was tried by the Kasirajan Gayathri and group and reported sensor **25** portraying the ON–OFF phenomena [108] (Fig. 9c). The detection ability of the probe was visible to the naked eye with the color changes from colorless to pale yellow. The reason for the color was predicted to be MLCT. The free probe absorption peak centered at 323 and 280 nm was changed considerably, with the new shoulder peak appearing at 380 nm. To further investigate the sensing behavior fluorescence study was conducted. Due to inhibition of the PET process, a redshift from 408 to 418 nm was observed after adding the zinc metals with intensity enhancement. The effect of pH was studied with the fluorescence intensity proved that the complex was stable and had maximum intensity at pH 7. The quantum yield of probe **25** changed from 0.019 to 0.214. The LOD and binding ratio was 2.9 nm and 1:1, respectively. The NMR and IR proved the coordinative mechanism. The zinc-**25** complex was reversible with the addition of EDTA. The live-cell imaging of the probe was conducted in N2A cells (Neuroblastoma from mouse). The fluorescence images showed an enhanced intensity after the incubation of.

It can be concluded that zinc can form five or six-membered stable complexes with the different probes. The presence of oxygen as the coordinating atom at position-1 and oxygen/nitrogen at position-7 was needed for the stable complex (Fig. 9d). All showed the enhancement of the intensity except **20** and **21**. The comparison of different parameters of the listed zinc ion sensor was tabulated in Table 5.

**Table 5:** Comparison of parameters of Zn^2+^ sensors

**Probe**	**Measured Signal**	**Stoichiometry**	**LOD**	**Quantum Yield of the probe**	**Real Samples**
**19**	UV–Vis/Fluorescence	–	–	0.057	–
**20**	UV–Vis/Fluorescence	1:1	4 × 10^–7^ M	0.26	–
**21**	UV–Vis/Fluorescence	1:1	0.12 µM	0.12	–
**22**	UV–Vis/Fluorescence	1:1	8.3 × 10^–7^ M	0.023	Neuron cell (SH-SY5Y) imaging
**23**	UV–Vis/Fluorescence	1:1	5.2 × 10^–7^ M	0.0028	Neuron cell (SH-SY5Y) imaging
**24**	UV–Vis/Fluorescence	1:1	1.02 µM	0.03	–
**25**	UV–Vis/Fluorescence	1:1	2.9 nm	0.019	N2A cell imaging

### Al^3+^

**Fig. 10:**
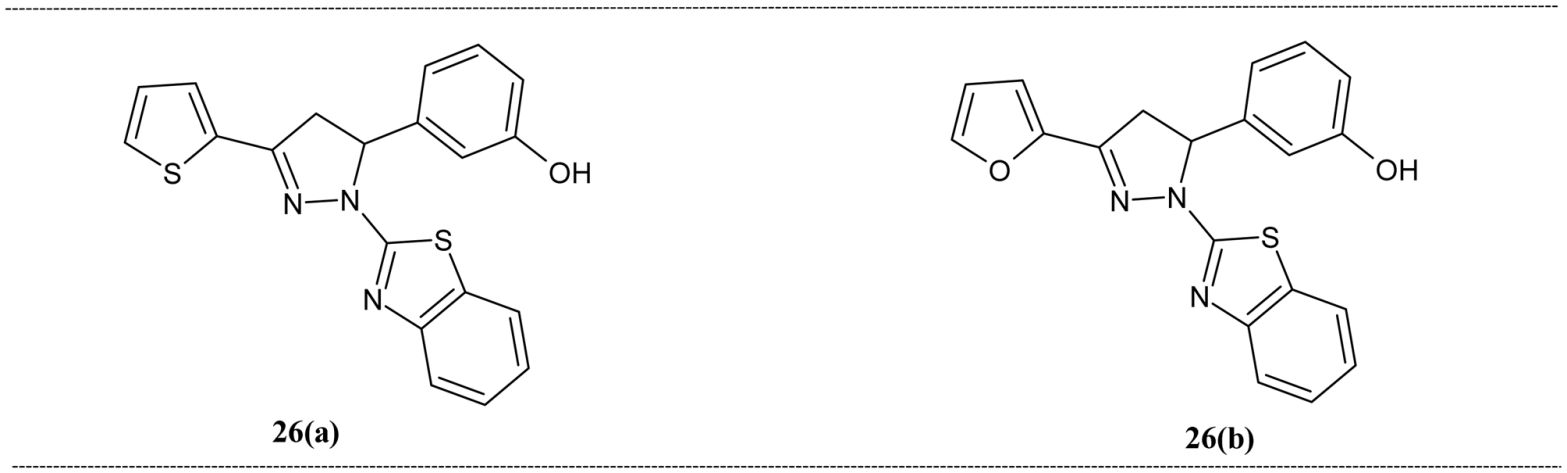
Pyrazoline-based Al^3+^ ion sensor decorated with benzothiazole moiety **26**

The furan and thiophene anchored pyrazoline derivatives were reported by Manjunath and Kannan for the Al^3+^ ion detection [109] (Fig. 10). The absorption spectra of receptors **26(a)** and **26(b)** were centered at 358 and 347 nm. This band shifted hypsochromically to 349 and 341 nm, attributed to the complex formation between receptor and Al^3+^. The fluorescence spectra of the probes had peaked at 447 and 436 nm for **26(a)** and **26(b),** respectively. In addition of the Al^3+^ ion quenching of the initially centered peak was observed for both the receptors. For **26(a),** the quantum yield decreased from 0.5 to 0.037, and for **26(b),** it dropped from 0.47 to 0.039. These probes displayed the bright blue fluorescence under UV light. The competition experiments showed the excellent selectivity of the receptors towards the Al^3+^ ion in the presence of other metal ions. The binding stoichiometry of the complexes was 1:1. The LOD of **26(a)** and **26(b)** was 8.92 × 10^–8^ M and 1.04 × 10^–7^ M, respectively. These values were found to be low but not lowest when compared to the previously reported probes.

## Multi ion Sensor

**Fig. 11:**
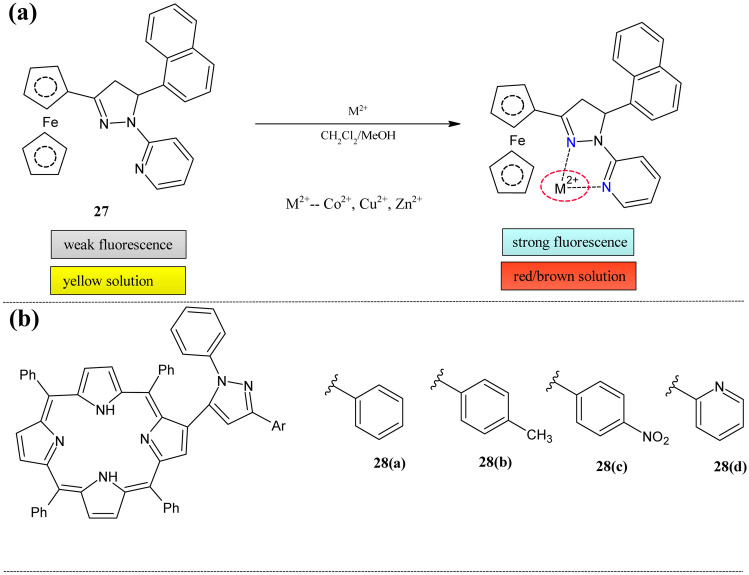
(**a**) Ferrocene based sensor **27** (**b**) Porphyrinic macrocycle **28**

Ferrocenes are efficient building blocks for the study of mixed-valence behavior and allow attachment of various functional groups with increasing charge transferability because of the stability in both oxidized and neutral states [110, 111]. Even though ferrocenes are known for their emission quenching behavior, luminescence properties are unaltered [112, 113]. Influenced by all these facts, Kumar et al. combined the ferrocene unit, fluorophore moiety, and coordinating unit(pyridyl pyrazoline) to detect and host the cations [114] (Fig. 11a). They reported the series of ferrocenyl pyridylpyrazoline compounds, among which **27** showed good sensing capacity in three different channels; fluorescent, colorimetric and electrochemical. The UV–Visible study showed that only Co^2+^, Zn^2+^, and Cu^2+^ were responsive, but other monovalent and divalent cations were unresponsive. The high energy (HE) band at 331 nm was redshifted on adding these ions with decreased absorption. The low energy (LE) band centered at 462 nm was also redshifted at about 4-13 nm. The stoichiometric ratio of the receptor to the metal was 1:1 for Zn^2+^, Cu^2+^ and 2:1 for Co^2+^. The ESI–MS confirmed these stoichiometries. In the fluorescence spectra, the band centered at 430 nm was redshifted to about 15 nm with a 4–ninefold increase in intensity. These compounds also showed suitable perturbation in redox couple with a cathodic shift in redox potential for the aforementioned cations.

The porphyrins and large macrocycles show excellent photophysical properties such as large excitation(> 400) and emission(> 600) wavelength, long stokes shift [115, 116], etc. The meso-position functionalized porphyrins are well known for their metal sensing abilities [117], but β-functionalized porphyrinic derivatives are less studied. To potentiate the sensing ability, pyrazoline moiety was decorated with the porphyrininc derivatives by Moura et al. for sensing metal ions like Hg^2+^, Cd^2+^, and Zn^2+^ [118] (Fig. 11b). The sensorial ability of the series of the pyrazole-porphyrin conjugates **28(a-d)** was tested using UV–visible and fluorescence spectroscopy. The absorption titration experiments of these conjugates with Zn^2+^ showed a slight bathochromic shift of the initial soret band with the formation of the new band at 660 nm. The fluorescence study of this metal showed the decreased intensity at 659 nm with the appearance of the new band at 608 nm on the addition of the zinc ions. The binding mode for **28(a-c)** was 1:2(M: L) and for **28(d)** 2:1(M: L). The absorption titration experiments of these aforementioned conjugates and Hg^2+^ were similar to the zinc. There was a more prominent red shift from 423 to 448 nm, with the new peak at 670 nm. The two isobestic points were observed at 438 and 510 nm. The excited state study of **28(a, b)** showed decreased intensity at 660 and 719 nm with the appearance of a new band at 704 nm. But for **28(c, d),** only quenching of the first two bands were observed. The ground state spectral titration of these probes with Cd^2+^ was similar to the previous two metals. But emission spectrum showed the more prominent quenching (≈55%) on the addition of the Cd^2+^ metal ions.

**Fig. 12:**
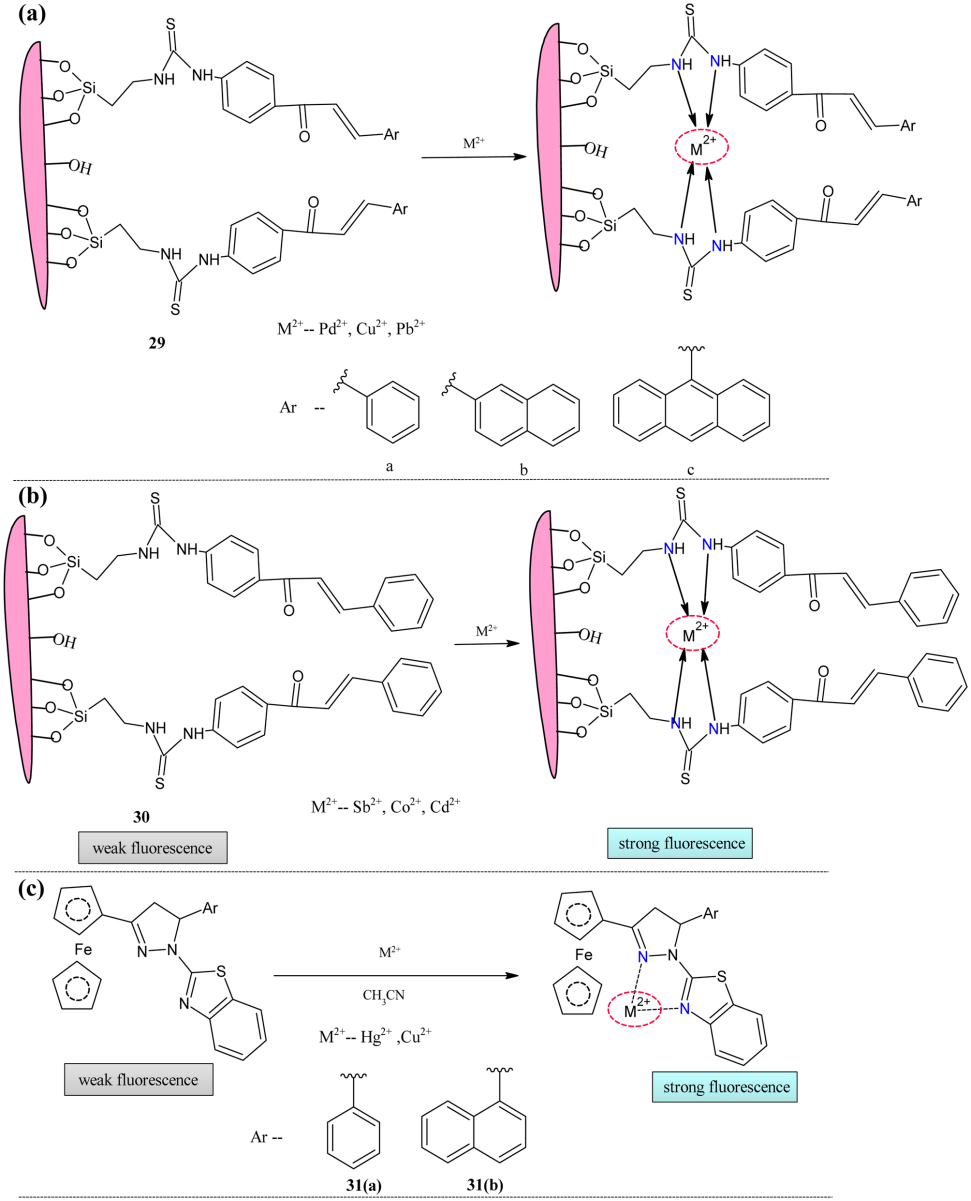
(**a**) (**b**) Nanosensor based on siloxy framework **29** and **30** (**c**) Ferrocene-Pyrazoline framework **31**

Fluorescent nanosensors are excellent metal ion sensors with good selectivity, sensitivity, and rapid response [119]. Recently more attention was paid to the development of mesoporous material solid-state sensors [120]. Among these mesoporous silicates have received much attention due to their excellent surface area, monodispersity, thermal stability, balanced pore size, etc. [110, 121]. Fayed et al. anchored the chalcones based on phenyl **29(a)**, naphthyl **29(b)**, and anthryl **29(c)** groups with the Korean Advanced Institute of Science and Technology(KIT-6) mesoporous silcates [114] (Fig. 12a). These probes were tested for their sensing ability towards the metal ions such as Pd^2+^, Cu^2+^, and Pb^2+^. In the absorption study, the **29** series compounds showed the increased absorption on the addition of the metal as mentioned above ions indicating the formation of donor–acceptor complexes. As emission intensity was more sensitive to the molecular structure, **29(a)** showed a significant enhancement in intensity than the other two probes because of the steric hindrance of **29(b)** and **29(c)**. The initially centered emission intensity at 405 nm was increased with a hypsochromic shift of 10 nm. The naked eye detection was possible with metal ions; color changed from pale yellow to yellow, pink, and cyan for Pd^2+^, Cu^2+^, and Pb^2^, respectively.

Nahass et al. reported similar nanocomposites for the toxic metal ion detection with the inclusion of new mesoporous material compared to the above [122] (Fig. 12b). The basic isothiocyanate moiety was anchored with two silicates, KIT-6 and Santa Barbara Amorphous (SBA-15). The sensing ability of the probe KIT-6 anchored chalcone **30** was tested with the Sb^2+^, Co^2+^, and Cd^2+^ metal ions by using absorption and emission spectroscopy. The color change was observed by adding the metal ions from pale yellow to cyan and colorless for Co^2+^ and Sb^2+^. No color change was observed with the addition of the cadmium metal ions since they had filled d and s orbitals. The fluorescence band centered at 405 nm was blue-shifted(≈10 nm) with the enhancement of intensity indicating the formation of donor–acceptor complexes.

The receptor attached ferrocene moieties were well known for their electrochemical detection of the metal ions. Its property of metal to ligand charge transfer was also used in the sensing field [123, 124]. Because of the excellent fluorescent properties of the pyrazoline moieties, it was integrated with the ferrocene group for cation sensing [114]. Kumar et al. explored this field by combing the five-membered benzothiazole moiety into the primary ferrocene group [125] (Fig. 12c). The cation sensing properties of probe **31** were monitored by measuring the optical and electrochemical properties. The properties were unchanged on the addition of the many divalent cations except for Cu^2+^ and Hg^2+^. The UV–visible titration of **31(a)** with Hg^2+^ metal ions showed the blue shift of HE bands to 330 nm(≈5 nm) with decreased intensity and redshift of LE band to 502 nm(≈52 nm) with increased intensity.

There was a new peak at 420 nm due to LMCT. Similar behavior was observed with the **31(b).** But LE band shifted to 504 nm, and the new band appeared at 414 nm. The color change from yellow to dark brown facilitated the naked-eye detection. Under the same experimental condition, the Cu^2+^ titration with **31(a)** showed a redshift of LE and HE band 456 nm(≈26 nm) 334 nm($\approx$ 6 nm), respectively. The intensity of the LE band was increased in contrast to the HE band. The **31(b)** HE band shifted to 342 nm with decreased intensity, and the LE band shifted to 484 nm with increased intensity. In **31(b)**, the HE bands redshifted to 342 nm with reduced intensity, and the LE band shifted to 484 nm with increased intensity. The color change was observed with the **31(b)** probe from yellow to red. The absence of the band beyond 600 nm indicates the absence of ferrocenium cation formed due to oxidation. The stoichiometric ratio of probes with Hg^2+^ was 1:1 and for Cu^2+^ 2:1(ligand: metal). Detection papers were prepared for the real-world application, which showed good color change when dipped in the corresponding metal solutions. The fluorescence also showed a good enhancement on the addition of the aforementioned cations. The 27 and 25-fold intensity enhancement was observed with **31(a)** and **31(b)**. The reversibility of the complex **31(a)**-Hg^2+^ was checked on the addition of the I^−^ ions. This addition decreased the intensity and again increased with negligible intensity loss. The proton NMR spectra proved the binding mechanism of the **31(a)**-Hg^2+^.

**Fig. 13:**
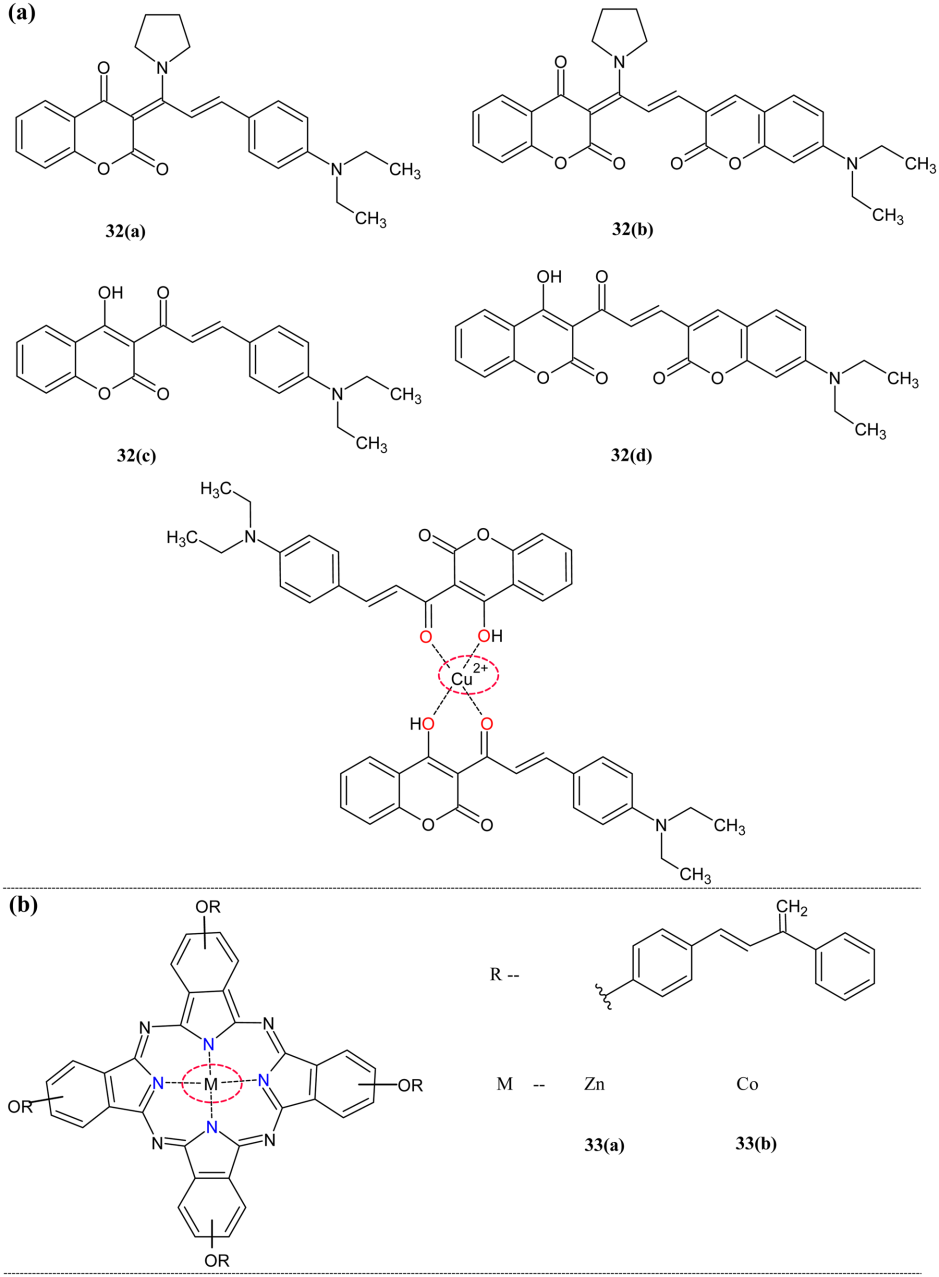
(**a**) Coumarinic chalcone derivative **32** (**b**) Metallophthalocyanine derivative **33**

Coumarinic derivatives are well known for their excellent photophysical properties, hence exploring the cation sensing field [126]. Shan et al. explored the area of coumarin-based chalcone derivatives to detect Cu^2+^ and CN^−^ [127] (Fig. 13a). The ground state study of **32(a)** and **32(b)** showed the decrease of absorption at 486 nm with an increased new band at 635 nm portrayed the complex formation. But in the case of **32(c)** and **32(d),** blue shift(484 nm) and redshift(525 nm) were observed, respectively. The fluorescence study of **32(a)** showed the turn-on response, and other probes obeyed the turn-off response. The Jobs plot revealed the stoichiometry of the complexes as 1:2(M: L) for **32(a-c)** and 1:1 for **32(d).** The DFT proved this ground-state data. The detection limit of **32(a)** was found to be 0.000059 µM. The selectivity of the 31 series compounds was studied in the presence of the other anions and cations. The **32(a-c)** showed the negligible absorption change but **32(d)** induced the absorption changes in the Fe^3+^ and Co^3+^ metal ions. This indicates the multi-ion sensing capacity of the probes. The color change was more prominent to differentiate the targeted metal ions from the other ions. The main advantage of this probe it could use as cyanide ion sensor too because study revealed the obivious absorption and fluorescence change on addition of the this.

Metallophthalocyanine shows good absorption in the UV–visible region, and hence its metal composites were widely applied in the field of photodynamics, photovoltaics, and dyes-catalyst chemistry [128–130]. The phthalocyanines combined chalcone would increase the solubility and give specific characteristics for the sensing behavior [131, 132]. The first metal phthalocyanines decorated chalcones **33(a)** and **33(b)** were monitored for their sensing capacity towards the cations Cu^2+^ and Fe^3+^ by Karaca et al*.* [127] (Fig. 13b). The UV–visible study showed that slight changes in the original spectra of the complexes. On Cu^2+^ titration, for **33(a)**, the band at 679 nm was redshifted to 722 nm, and a new band appeared at 492 nm, but **33(b)** Q-band at 670 nm was shifted to 730 nm with the appearance of the new peak at 513 nm. The Fe^3+^ titration of **33(a)** and **33(b)** showed similar changes, i.e., the redshift of band 679 nm to 721 nm. This promotes the phthalocyanine-derived chalcone hybrids' metal ion sensing ability.

**Fig. 14:**
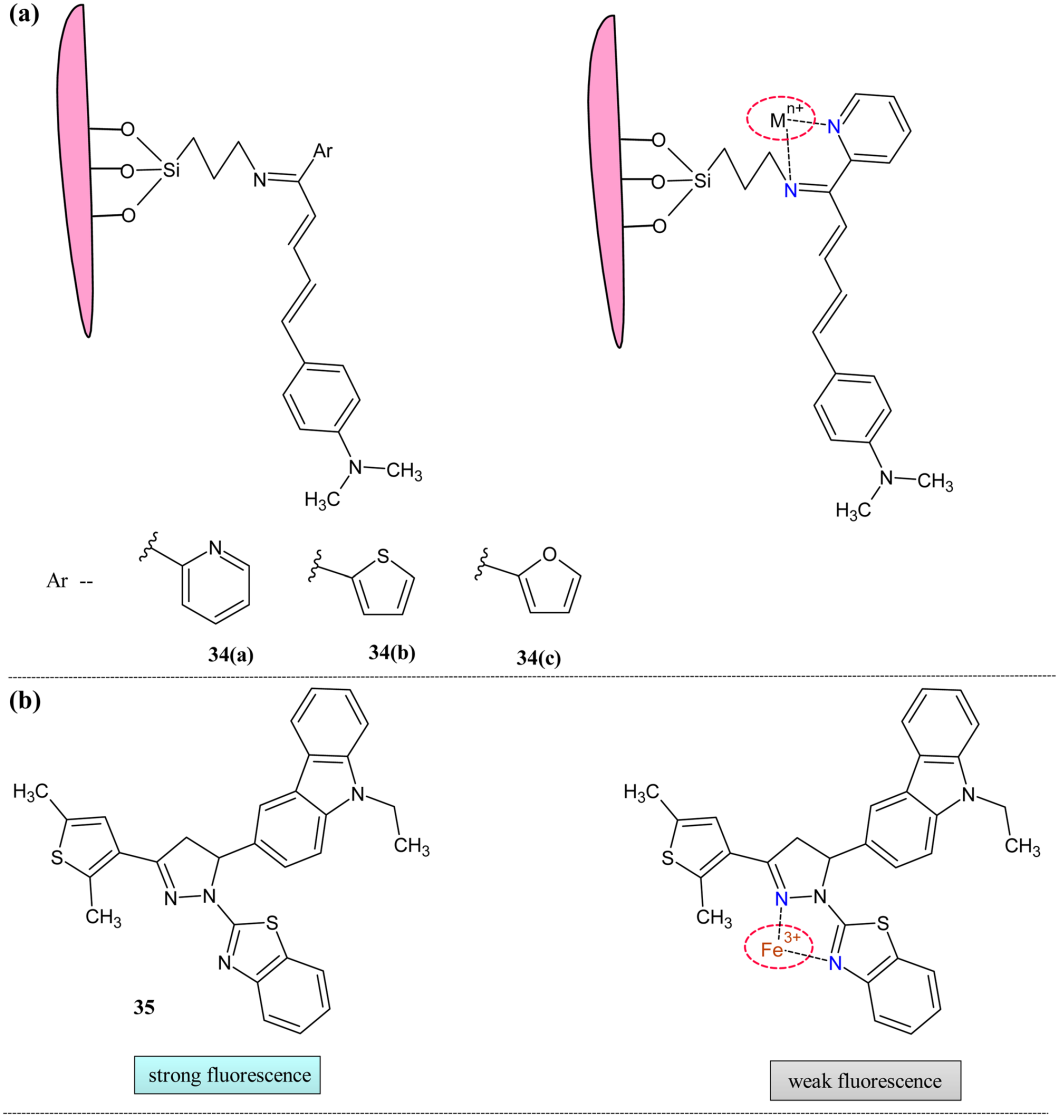
(**a**) SBA-16 anchored nanosensor **34**, (**b**) Structures of triaryl pyrazoline derivative during sensing **35**

Fluorescent nanosensors are mainstreamed because of quick and accurate detection, qualitative and quantitative analysis, and selectivity; hence, they have the earmark of cation sensors [133–135]. To explore the field of mesoporous decorated chalcone derivative, Nahass and Fayed reported SBA-16 anchored chalcone hybrids to detect toxic metals [136] (Fig. 14a). The absorption study showed an appreciable increase in the absorption intensity with the appearance of the new peaks on the addition of the di and trivalent metal ions such as Hg^2+^, Mn^2+^, Cd^2+^, Fe^3+^, Cu^2+^, Co^2+^, Zn^2+^, and Ni^2+^. For **34(a),** the original band centered at 351 nm get redshifted to a few nanometers (≈9 nm) on the addition of the aforementioned cations. Similarly, for **34(b)** and **34(c),** redshift was from 348 and 357 nm, respectively. The binding constant of the nanosensors was calculated from the Benesi-Hildebrand equation. Its value revealed that **34(a)** had better banding ability with the sensed metal ions than the other two because of the presence of nitrogen as a heteroatom. In the fluorescence study, all the probes showed a pronounced decrease in the fluorescence intensity on the addition of the targeted metal ions. In addition to this, blue or red shift was observed for band centered at 462,453 and 504 nm for **34(c)**, **34(b)** and **34(a)** respectively. As a representative example, Cd^2+^ titration showed the blue shift band at 442 nm (29.4% quenching) for **34(a)**, red shift band at 457 nm (56.7% quenching) for **34(b)** and redshifted band at 512 nm (10.4% quenching) for **34(c)**. The sensors showed obvious color changes in presence of the metal which can be discerned by the bare eye.

Triaryl pyrazoline derivatives are earmarked for their excellent fluorescence properties due to hindering of the double bond [137] (Fig. 14b). In one such attempt, Asiri et al. reported probe **35** to detect metal ions such as Fe^2+^, Fe^3+^, and Cu^2+^ [138]. The photophysical behavior of carbazole containing pyrazole in different solvents was studied; it showed the probe as an excellent solvatochromic dye of the pyrazoline family. The fluorescence study of the compound showed good emission at 335 nm in DMF/water (9:1). In addition to the Fe^2+^, Fe^3+^ and Cu^2+^ intensity quenched of about threefold,sevenfold, and fivefold respectively. In addition to the Fe^3+^ ions, the color change was observed from blue to brown. The quantum was decreased from 0.57 to 0.18on the addition of ferric ions. The Jobs plot revealed the 1:1 stoichiometry of the complexes. The chelation enhanced quenching causes decreased intensity on the bonding of metal ions with pyrazoline and benzothiazole moiety. So, it indicates that this probe can be used as good fluorescent for the Fe^3+^ ion detection.

Compared with the single ion sensors, multi-ion sensors were practically applicable to the infield sensing purpose. Different varieties of chalcone derivatives were reported as a sensor for both di and trivalent metal ions. We can observe that there was no focus on the regeneration of the original probe, and also, none of the sensors were tested for the real samples. So, the exploration of this field is still expected. The parameters of the probes are listed in Table 6.

**Table 6:** Comparison of parameters of multi-ion sensors

**Probe**	**Measured Signal**	**Metal ions sensed**	**Type of probe**	**Reversibility**	**Real Samples**
**27**	UV–Vis/Fluorescence	Co^2+^, Zn^2+^, Cu^2+^	Ferrocenyl-Pyrazoline	–	–
**28**	UV–Vis/Fluorescence	Hg^2+^, Cd^2+^, Zn^2+^	Porphyrin-Pyrazoline	–	–
**29**	UV–Vis/Fluorescence	Pd^2+^, Cu^2+^, Pb^2+^	Nanosensor	–	–
**30**	UV–Vis/Fluorescence	Sb^2+^, Co^2+^, Cd^2+^	Nanosensor	–	–
**31**	UV–Vis/Fluorescence	Cu^2+^, Hg^2+^	Ferrocene-Pyrazoine	I^−^	Dip strip
**32**	UV–Vis/Fluorescence	Cu^2+^, CN^−^	Coumarinic chalcone derivative	–	–
**33**	UV–Visible	Cu^2+^, Fe^3+^	Metallophthalocyanine-chalcone	–	–
**34**	UV–Vis/Fluorescence	Hg^2+^, Mn^2+^, Cd^2+^, Fe^3+^, Cu^2+^, Co^2+^, Zn^2+^, Ni^2+^	Nanosensor	–	–
**35**	Fluorescence	Fe^2+^, Fe^3+^, Cu^2+^	Pyrazoline	–	–

## Conclusion and Perspectives

To sum up, in this review, we stacked up all available literature for chalcone-derived metal ion sensors. The fluorescence and absorption spectra become the basis for the sensing. The fluorescence phenomenon was often used for sensing because of its sensitivity and more intense interaction of light and matter than the rapid absorption. The turn-on sensing gives more exposure to the sensing field than turn-off because of its low detection limits and bright background. The ratiometric probes also have significant advantages as they may reduce the variation caused by the environmental condition, the efficacy of the instrument, and concentration. It can be seen from the study that there was more focus on the pyrazoline derivative of chalcone than compared to any other chalcone analogous. This may be because of the structural diversification of the pyrazoline. The tieing up of the three heterocyclic aryl groups into the pyrazoline core will introduce the coordinating site to the incoming cations, and also it may enhance or quench the intensity of emission. The most frequently attached moiety to the nitrogen of pyrazoline was the benzothiazole group, as it may provide coordinating site results in a stable five-membered ring with the cations. Similarly, the third carbon of the pyrazoline ring should possess a hetero substituted aryl group at the ortho position; it results in the stable five-membered structure on coordination. Besides the pyrazoline derivative, many peculiar groups were attached to the basic chalcone skeleton to induce the emission behavior. Few such attempts were macrolide-chalcone derivative, isatin chalcone-siloxy framework, aza-BODIPY based chalcone, organosilicon based chalcone, mesoporous siloxy group decorated nanosensor, etc.

In the field of single ion sensors, there was more focus on copper and zinc ion sensing. The more interesting point was that all the zinc ion sensors were pyrazoline-based probes. China was more focused on cation sensing than other countries then followed by India. Similarly, if we consider the yearly contribution, in 2013, there was more attention to this field. Nonetheless, many chalcone-derived sensors were reported; still, there is a need to focus light on the other type of this derivative. As most of the derivatives were tasted in the non-aqueous media, there is a need to develop water-soluble probes. These water-soluble probes make a more feasible real-world application of the probes for the human betterment of life. We expect this field needs more exploration because of chalcones' quick and simple synthetic methods, good pharmacological properties resulting in eco-friendly probes to the environment, good sensitivity, and selectivity towards the metal ions. We believe that with new synthetic strategies, novel chalcone-derived probes will be reported, and it will find more relevant applications in the analytical field for the betterment of the biosystems.

## Data Availability

The data that support the findings of this study are available from the corresponding author upon reasonable request.
